# A comparison of the morphological and biochemical characteristics of *Chlorella sorokiniana* and *Chlorella zofingiensis* cultured under photoautotrophic and mixotrophic conditions

**DOI:** 10.7717/peerj.3473

**Published:** 2017-09-08

**Authors:** Siti Nor Ani Azaman, Norio Nagao, Fatimah M. Yusoff, Sheau Wei Tan, Swee Keong Yeap

**Affiliations:** 1 Laboratory of Immunotherapeutics and Vaccines, Institute of Bioscience, Universiti Putra Malaysia, Serdang, Selangor, Malaysia; 2 Laboratory of Marine Biotechnology, Institute of Bioscience, Universiti Putra Malaysia, Serdang, Selangor, Malaysia; 3 China-ASEAN College of Marine Sciences, Xiamen University Malaysia, Sepang, Selangor, Malaysia

**Keywords:** Pigments content, *Chlorella zofingiensis*, Total phenolic content, Antioxidant activity, *Chlorella sorokiniana*, Carotenoid content

## Abstract

The responses of two species of microalgae, *Chlorella sorokiniana* and *Chlorella zofingiensis*, were compared regarding their morphological and biochemical properties under photoautotrophic and mixotrophic conditions. These microalgae were cultured under both conditions, and their crude ethanolic extracts were examined for their pigment and total phenolic contents. In addition, the microalgae’s antioxidant activities were determined using a DPPH radical scavenging assay and a ferric reducing antioxidant power (FRAP) assay. Both strains showed increases in cell size due to the accumulation of lipid bodies and other cell contents, especially carotenoids, under the mixotrophic condition. Notably, reductions in phenolic and chlorophyll contents were observed to be associated with lower antioxidant activity. *C. zofingiensis* compared with *C. sorokiniana*, demonstrated higher antioxidant activity and carotenoid content. This study showed that different species of microalgae responded differently to varying conditions by producing different types of metabolites, as evidenced by the production of higher levels of phenolic compounds under the photoautotrophic condition and the production of the same levels of carotenoids under both photoautotrophic and mixotrophic conditions.

## Introduction

Microalgae have been identified as good sources of bioactive metabolites, including polyphenol, vitamins, lipids for use as biofuels and proteins, warranting the sustainable utilisation of microalgae for energy, food and health applications (Chacón-Lee & González-Mariño, 2010; Mostafa, 2012). The chemical compounds synthesised by microalgae are usually classified into primary and secondary metabolites based on their chemical functional groups and biosynthetic origins (Kumar, Dasgupta & Das, 2014). The relative contents of various metabolites in different varieties of microalgae are fairly similar under normal condition of growth. However, these contents change under sub-optimal conditions (Hu, 2004; Solovchenko et al., 2008; Juneja, Ceballos & Murthy, 2013). Changes in various environmental factors, such as temperature, light, pH and nutrients levels, can affect many cellular activities, including photosynthesis, growth efficiency, cellular metabolism and cell composition. For example, during photosynthesis, the contents of pigments such as chlorophyll a and chlorophyll b, which function as light-harvesting antennae in the main reaction centre (Lodish et al., 2000; Masojídek, Koblízek & Torzillo, 2004), and primary carotenoids might decrease in response to high light intensity; by contrast, secondary carotenoids that serve as photoprotective agents increase under these conditions (Hu, 2004; Darko et al., 2014). Primary carotenoids, such as β-carotene, lutein and zeaxanthin, usually accumulate in the chloroplast, whereas secondary carotenoids, such as astaxanthin, canthaxanthin and adonixanthin are found in lipid bodies outside the chloroplast (Grünewald, Hirschberg & Hagen, 2001). Primary metabolites are usually produced to maintain the physical integrity and are key for the survival of cells, whereas secondary metabolites are not vital for cell survival but instead maintain the proper functions of all physiological systems. Both primary and secondary metabolite pools consist of antioxidants such as phenols, carotenoids, terpenoids and flavonoid derivatives (Cardozo et al., 2007). Depending on the ability to handle various growth conditions, different strains of microalgae produce different metabolites to increase their chances of survival (Skjanes, Rebours & Lindblad, 2013; De Morais et al., 2015).

Carotenoids are among the best-known antioxidants originating from microalgae and play an important role in protecting the microalgal system. These pigment molecules directly quench singlet oxygen, thereby preventing free radical reactions (Vachali, Bhosale & Bernstein, 2012; Safafar et al., 2015). Previous studies have revealed that carotenoids contribute significantly to the total antioxidant capacity of microalgae (Takaichi, 2011; Goiris et al., 2012). Thus, microalgae have become an alternative source of carotenoids such as astaxanthin from *Haematococcus* and β-carotene from *Dunaliella*) that are used in the food and pharmaceutical industries (Spolaore et al., 2006). Other important antioxidant compounds that can be obtained from microalgae are the phenolic compounds, which consist of several classes of flavonoids and non-flavonoids. These compounds also protect the microalgae from damage through single electron transfer and hydrogen atom transfer (Ndhlala, Moyo & Staden, 2010; Leopoldini, Russo & Toscano, 2011). Although little is known approximately the presence of phenolic compounds in microalgae, several studies have demonstrated that they contribute significantly to the antioxidant capacity of certain species of microalgae (Hajimahmoodi et al., 2010; Goiris et al., 2012; Safafar et al., 2015).

Due to its specific characteristics, *Chlorella* spp. have become one of the most heavily researched microalgal groups by scientists due to their characteristics, including a high nutritional value in terms of natural antioxidants (Matsukawa et al., 2000; Rodriguez-Garcia & Guil-Guerrero, 2008; Hajimahmoodi et al., 2010; Sawant et al., 2014), high productivity in terms of lipid and carbohydrate contents (Del Campo et al., 2004; Goiris et al., 2012; Zhu et al., 2014; Goiris et al., 2015), and a thick cell wall that protects their nutrient contents (Iwamoto, 2004). Moreover, previous studies have demonstrated that the composition of microalgae can be controlled by changing the growth medium and by culturing under different growth conditions. For example, one study showed that different physiological and biochemical properties are produced by the same microalgae under different growth conditions (Iwamoto, 2004). Moreover, the colour of *Chlorella* has been shown to change from green to red or yellowish based on the pigments produced when grown under different conditions (Del Campo et al., 2004; Ip, Wong & Chen, 2004; Ip & Chen, 2005; Cordero et al., 2011). Different cultivation conditions may also affect both the production of metabolites and the processing cost. Most studies have reported that compared to autotrophic or heterotrophic conditions, mixotrophic condition are more advantageous in terms of growth rate and productivity (Yang, Hua & Shimizu, 2000; Ip, Wong & Chen, 2004; Liang, Sarkany & Cui, 2009; Shetty & Sibi, 2015). However, despite the indisputable advantages, mixotrophic culture conditions have been comparatively underutilised in commercial production (Del Campo, García-González & Guerrero, 2007). Although some criticism of the photoautotrophic condition exist (Jorquera et al., 2010), such conditions are still most commonly utilised for the large-scale cultivation of microalgae for use in commercial applications (Mimouni et al., 2012). It has also been reported that photoautotrophic conditions are better than mixotrophic conditions for the production of certain metabolites (Abreu et al., 2012).

In this study, two species of *Chlorella*, *C. sorokiniana* and *C. zofingiensis*, were selected for their abilities to produce valuable metabolites that have potential applications in the pharmaceutical and health industries (Matsukawa et al., 2000; Brányiková et al., 2011; Liu et al., 2014). These strains are characterised by their high growth rates and high tolerances of various temperatures used during culture. These characteristics are expected to offer significant advantages for use in large-scale production bioreactors. Although *Chlorella* spp. are frequently consumed as a health supplement, most studies of *C. sorokiniana* and *C. zofingiensis* have focused mainly on the profiling of their biochemical contents such as lipids and carotenoids, and less on their antioxidant capacities (Del Campo et al., 2004; Ip & Chen, 2005; Wang & Chen, 2008; Liu et al., 2014). To date, most studies microalgae have focused on the production of mass and metabolites under different cultivation conditions, and research into the morphological and ultrastructural changes of microalgae under various conditions is still lacking. Thus, in the present study, we gathered and evaluated information on the morphological and biochemical characteristics of *C. sorokiniana* and *C. zofingiensis* grown under photoautotrophic and mixotrophic conditions while focusing primarily on their antioxidant activities, which were assessed using DPPH radical scavenging and ferric-reducing antioxidant power (FRAP) assays.

## Materials and Methods

### Microalgal culture

The *Chlorella* species examined in this study were obtained from the Marine Biotechnology Laboratory at the Faculty of Agriculture, Universiti Putra Malaysia, which had originally been obtained from UTEX and NIES (the culture collections of algae at the University of Texas, USA and the National Institute of Environmental Studies, Japan). The strains examined were *Chlorella sorokiniana* (NIES-2168) and *Chlorella zofingiensis* (ATCC30412). The pre-culture microalgae were inoculated with 10% (vol/vol) of an exponentially growing culture in Bold’s Basal Medium (BBM) at a total volume of 200 mL (in 2 flasks of 100 mL each) at 27 °C. The microalgal cultures were grown under continuous light with an intensity of approximately 10 µmol photons m^−1^ s^−1^ with a shaking speed of 30 rpm.

**Figure 1:**
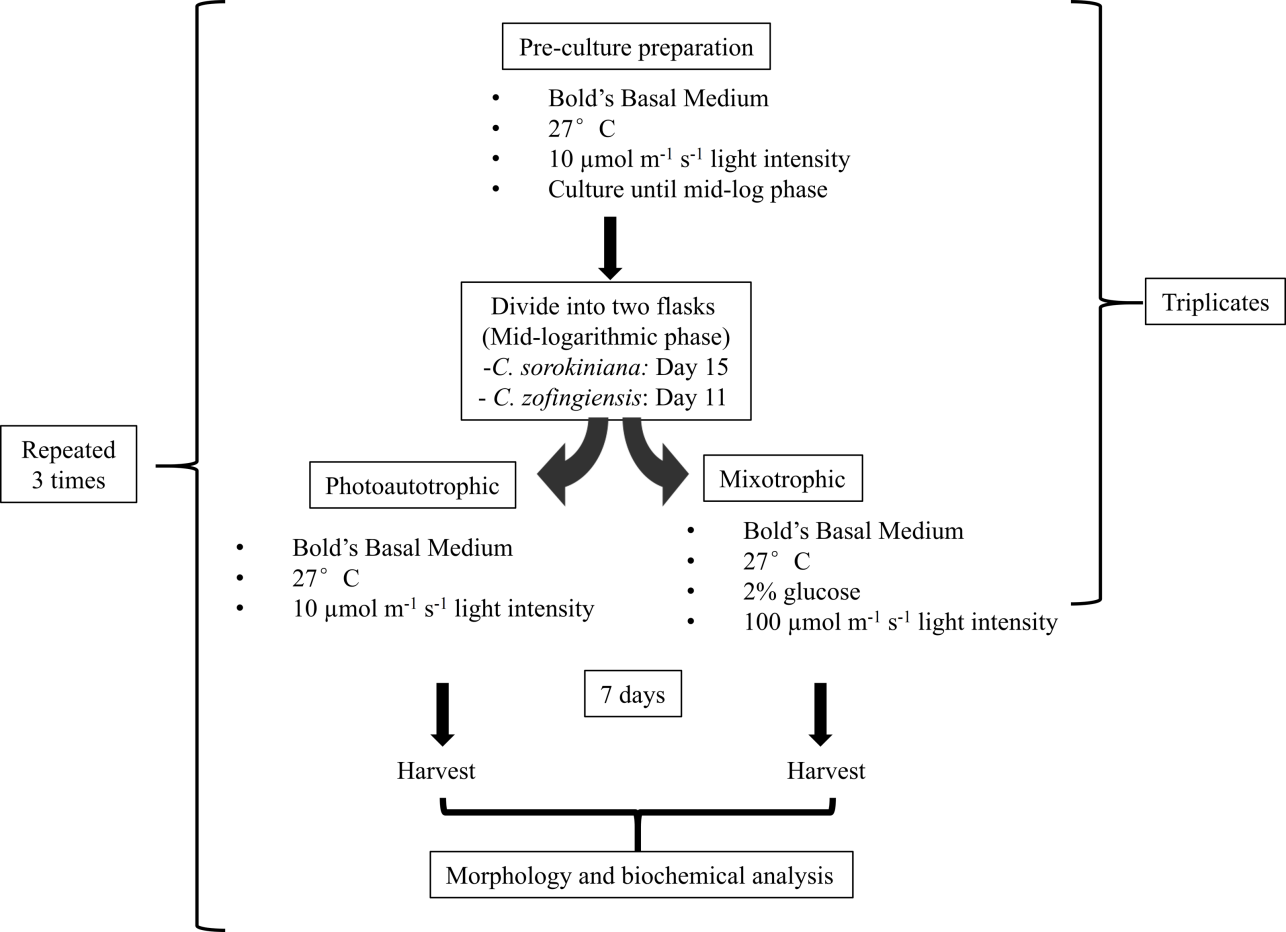
Experimental workflow used in this study.

Triplicates of pre-culture microalgae were allowed to grow until the mid-logarithmic phase, reaching approximately 8–10 × 10^6^ cells/mL on day 11 and 2.5–3.0 × 10^6^ cells/mL on day 15 for *C. zofingiensis* and *C. sorokiniana*, respectively. Then, both microalgae cultures were further divided into two flasks, and each flask contained 100 mL of 2 × 10^6^ cells/mL. One flask of cells was cultured under photoautotrophic condition and the other was cultured under mixotrophic condition. The same culture sources were used to reduce the variability of the cultures in subsequent comparisons. The photoautotrophic condition was the same as the pre-culture condition, whereas the mixotrophic culture condition consisted of a higher light intensity than the photoautotrophic condition and the addition of glucose. Generally, mixotrophic conditions trigger changes in the microalgae that enhance the production of metabolites, as indicated by colour changes of the culture. Several approaches for imposing changes in the colour of the microalgae cultures were tested (Latasa & Berdalet, 1994; Chokshi et al., 2015), and the approach utilised in this study was based on a strategy by Ip, Wong & Chen (2004), with minor modifications. To induce colour changes in the microalgae, the cultures were transferred to a medium light intensity at 100 µmol photons m^−1^ s^−1^ with the addition of 2% glucose. Microalgae cultured under both conditions were allowed to grow for 7 days once the mixotrophic condition was initiated at the mid-logarithmic phase. The experiments were conducted in a shaking incubator, and a conical flask was used as the growth chamber; the white fluorescence light source was located above the cultures. All experiments were repeated independently in triplicate. The microalgae were harvested by separating the pellet from the medium by centrifugation at 10,000 rpm for 10 min. The pellet was then flash-frozen using liquid nitrogen and stored storage at −20 °C prior to use. All experiments were repeated three times (Fig. 1).

### Morphological observation using light and electron microscopy

The microalgae were examined under alight microscope (Olympus FSX100, Japan) using a bright field objective lens. For transmission electron microscopy, the microalgae were fixed in 4% glutaraldehyde for 12 h at 4 °C. The fixed cells were then washed three times, 10 min each, using 0.1 M sodium cacodylate buffer. After post-fixation in 1% osmium tetroxide for 2 h at 4 °C, the cells were washed again and dehydrated in a serial dilution of acetone (35% to 100%) for 15 min. The cells were then infiltrated, and the beam capsule was filled with a resin mixture, and polymerization occurred in a 67 °C oven. Semi-thin sections (1 µm thick) were cut using an ultramicrotome (Leica-Reichert Ultracut S, Austria). The sections were then stained with toluidine blue and viewed under a light microscope to select the region of interest prior to ultrathin sectioning. Ultrathin sections were cut and mounted onto 200-mesh copper grids and stained with uranyl acetate and lead citrate for 10 min. The stained sections were finally examined using a transmission electron microscope (TEM) (Hitachi H7100, Japan) at 80 kV.

For scanning electron microscopy, the microalgae were treated as for the transmission electron microscopy until the serial dehydration step with acetone. Subsequently, the cells were coated with albumin on an aluminium foil with a diameter of 1 cm. The cells were then transferred into a specimen basket for the critical point drying step for approximately 30 min. After drying, the cells were mounted onto the specimen stub using double-sided tape or colloidal silver. The cells were then coated with gold particles using a sputter coater and examined under a scanning electron microscope (SEM) (JEOL, JSM-6400) at 15 kV.

### Extract preparation

The ethanol extract of microalgae was obtained according to Hemalatha et al. (2013) and Saranya et al. (2014). Briefly, the harvested microalgae were ground with a mortar and pestle. A 0.2 g sample of ground microalgae was extracted for 24 h in 10 mL ethanol at room temperature. The extraction was repeated twice and the extract was filtered through Whatmann filter paper. Each filtrate was concentrated to dryness under reduced pressure using a SpeedVac Concentrator 5310 (Eppendorf, Germany). Finally, the dry extracts were lyophilised and stored at −20 °C for further analysis.

### Determination of pigment content

The pigment contents of the microalgae were determined using a method described by Lichtenthaler & Buschmann (2001). Briefly, the extracted sample was dissolved in 95% ethanol, filtered through two layers of cheese cloths and centrifuged at 2,500 rpm for 10 min. The supernatant was separated and the absorbance was measured at 400–700 nm on a UV/Vis spectrophotometer (Pharmacia Ultrospec 3000 pro). According to Lichtenthaler & Buschmann (2001), chlorophyll a, chlorophyll b, and total carotene show maximum absorbance at 664 nm, 648 nm and 470 nm, respectively. The concentrations of these pigments were calculated according to the following formula:

\begin{eqnarray*}& & {C}_{a}(\mathrm{\mu }\mathrm{g/ mL})=13.36\;{A}_{664.1}-5.19\;{A}_{648.6} \end{eqnarray*}

\begin{eqnarray*}& & {C}_{b}(\mathrm{\mu }\mathrm{g/ mL})=27.43\;{A}_{648}-8.12\;{A}_{664.1} \end{eqnarray*}

\begin{eqnarray*}& & {C}_{(x+c)}(\mathrm{\mu }\mathrm{g/ mL})=(1,000\;{A}_{470}-2.13{C}_{a}-97.64\;{C}_{b})/209 \end{eqnarray*}

where *C*_*a*_ is chlorophyll a, *C*_*b*_ is chlorophyll b and *C*_(*x*+*c*)_ is total carotene.

### Total phenolic content (TPC)

The phenolic contents of the ethanolic extracts were estimated using the Folin-Ciocalteau method (Taga, Miller & Pratt, 1984). An aliquot sample (100 µL) was mixed with 2.0 mL of 2% Na_2_CO_3_ and allowed to stand for 2 min at room temperature. After incubation, 100 µL of 50% Folin-Ciocalteau’s phenol reagent was added and the reaction mixture was mixed thoroughly and allowed to stand for 30 min at room temperature in the dark. The absorbance of each sample solutions was measured at 750 nm using a UV/VIS spectrophotometer. The blank consisted of all reagents and solvents without samples. Gallic acid was used as a positive control and was diluted in concentrations ranging from 1.0 mg/mL to 0.001 mg/mL. The phenolic contents of the samples were expressed as the gallic acid equivalent (GAE) per mg dry weight of sample. The results are presented as the means of triplicate experiments ± standard deviation.

### DPPH radical scavenging assay

Free radical scavenging activity was measured using 2,2-diphenyl-1-picrylhydrazyl (DPPH) according to the method described by Cox, Abu-Ghannam & Gupta (2010). Briefly, a 2.0 mL aliquot of the test sample was added to 2.0 mL of 0.16 mM DPPH methanolic solution. The mixture was vortexed for 1 min and incubated at room temperature for 30 min in the dark. The absorbance of the sample solution was measured at 517 nm using a UV/VIS spectrophotometer. The ability to scavenge the DPPH radical activity was calculated using the following equation:

\begin{eqnarray*} \left[ 1- \left( \frac{\text{Sample}-\text{Sampleblank}}{\mathrm{Control}} \right) \right] \times 100 \end{eqnarray*}

where *sample* is the absorbance of the test sample containing the DPPH solution, sample blank is the absorbance of the sample without the DPPH solution, and control is the absorbance of the DPPH solution without the sample. In this study, ascorbic acid was used as a positive control. The results are presented as the means of triplicate experiments ± standard deviation.

### Ferric reducing antioxidant power assay

The ferric-reducing antioxidant power (FRAP) assay of the ethanolic extract was carried out according to Hajimahmoodi et al. (2010). Briefly, the FRAP reagent containing 5 mL of a 10 mM TPTZ (2,4,6-tripyridyl-S-triazine) solution in 40 mM HCl plus 5 mL of 20 mM FeCl_3_ and 50 mL of 0.3 M acetate buffer (pH 3.6) was freshly prepared and incubated at 37 °C. A 100 µL extract of each sample was mixed with the FRAP reagent and incubated at 37 °C for 10 min before being measured at 593 nm. When necessary, the extracted samples were appropriately diluted with ethanol. A known concentration of ascorbic acid was used as a positive control, and the final results were expressed as the micromolar ascorbic acid equivalent (µM AAE) per mg dry weight of sample. The results are presented as the means of triplicate experiments ± standard deviation.

**Figure 2:**
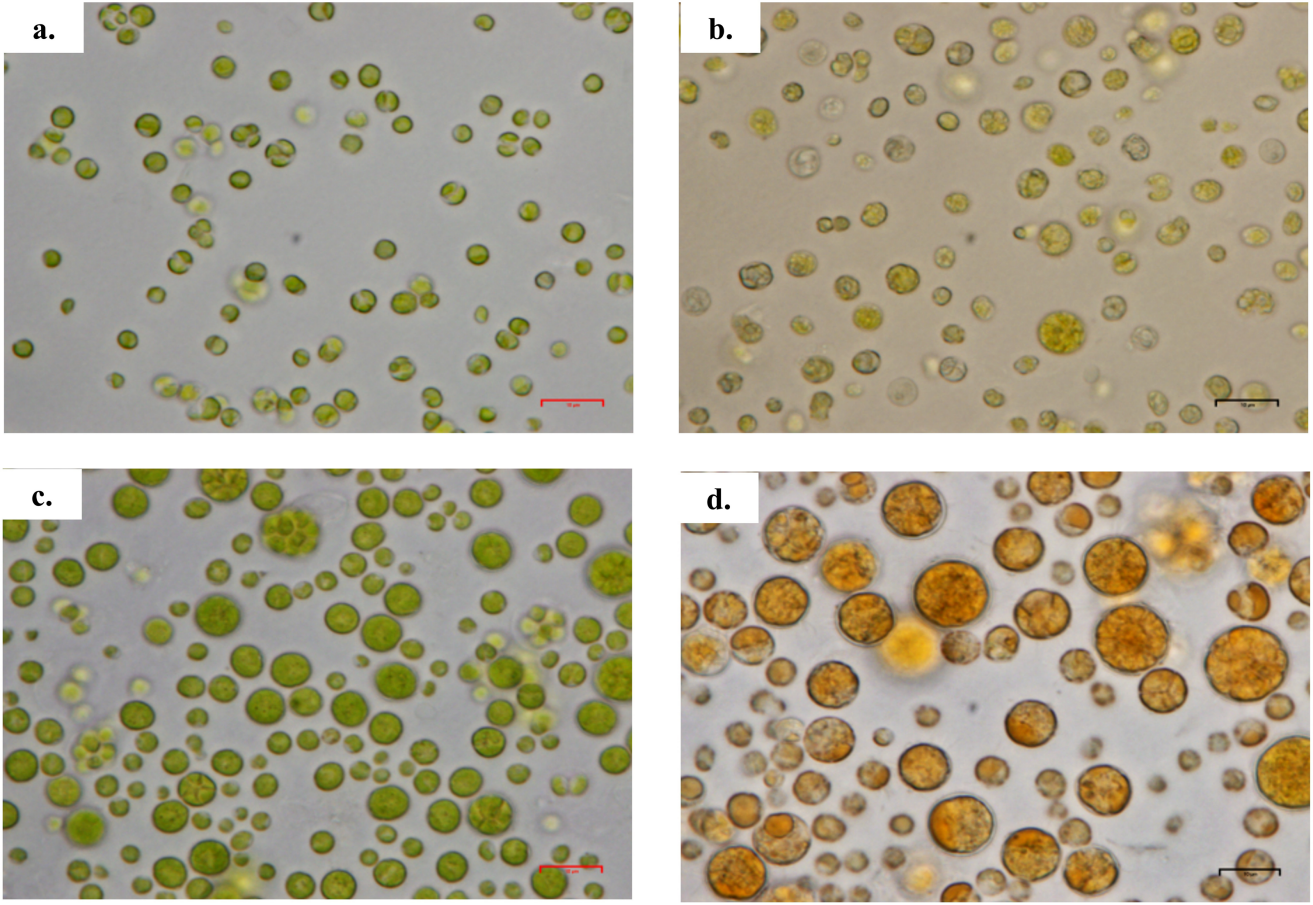
Morphology of *Chlorella* spp. under light microscope. (A) *Chlorella sorokiniana* in photoautotrophic condition, (B) *Chlorella sorokiniana* in mixotrophic condition, (C) *Chlorella zofingiensis* in photoautotrophic condition and (D) *Chlorella zofingiensis* in mixotrophic condition. Scale bar 10 µm.

## Results and Discussion

### Morphological changes

**Figure 3:**
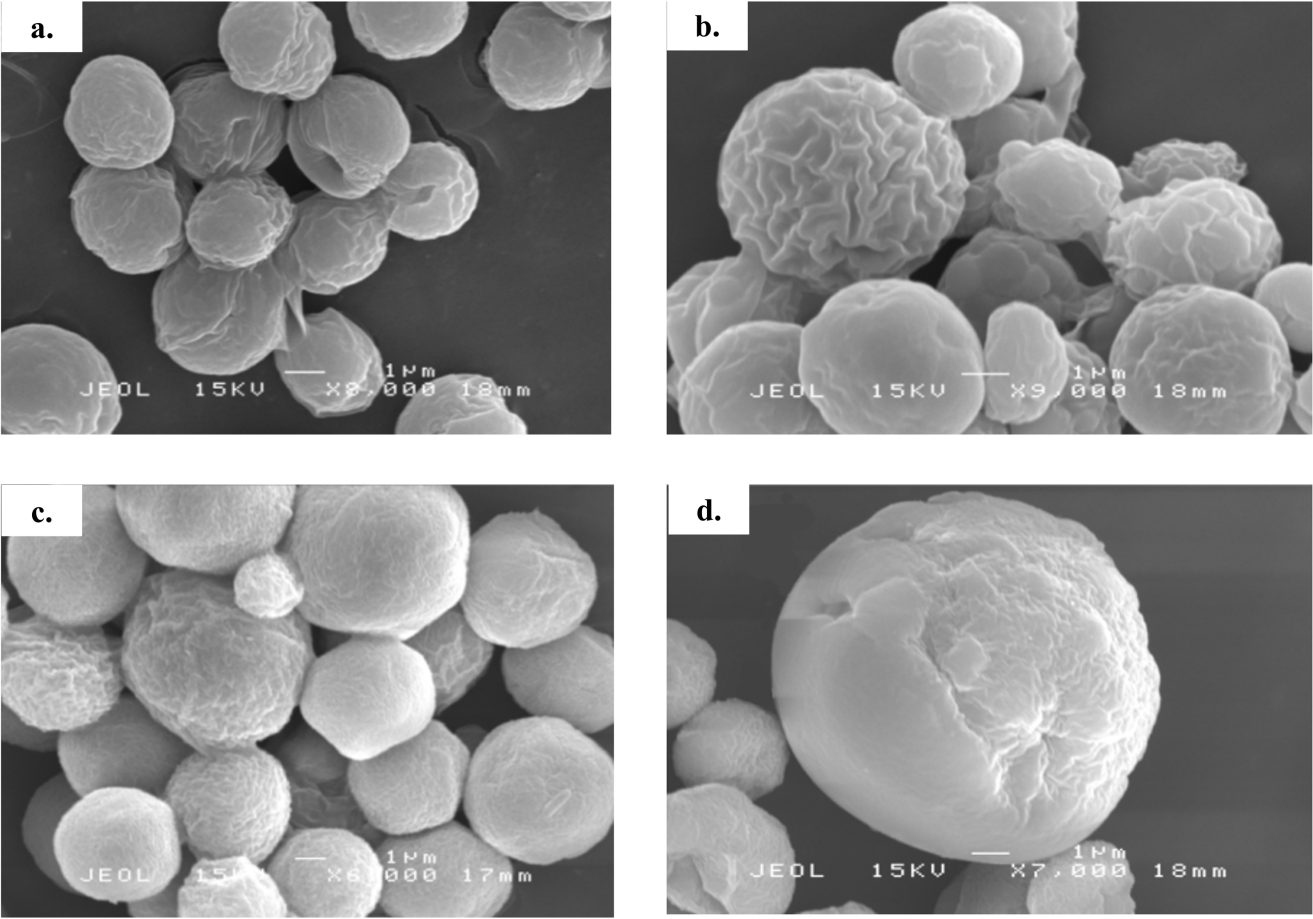
Scanning electron micrograph of *Chlorella* spp. under photoautotrophic and mixotrophic conditions. (A) *C. sorokiniana* in photoautotrophic condition, (B) *C. sorokiniana* in mixotrophic condition, (C) *C. zofingiensis* in photoautotrophic condition, and (D) *C. zofingiensis* in mixotrophic condition.

**Figure 4:**
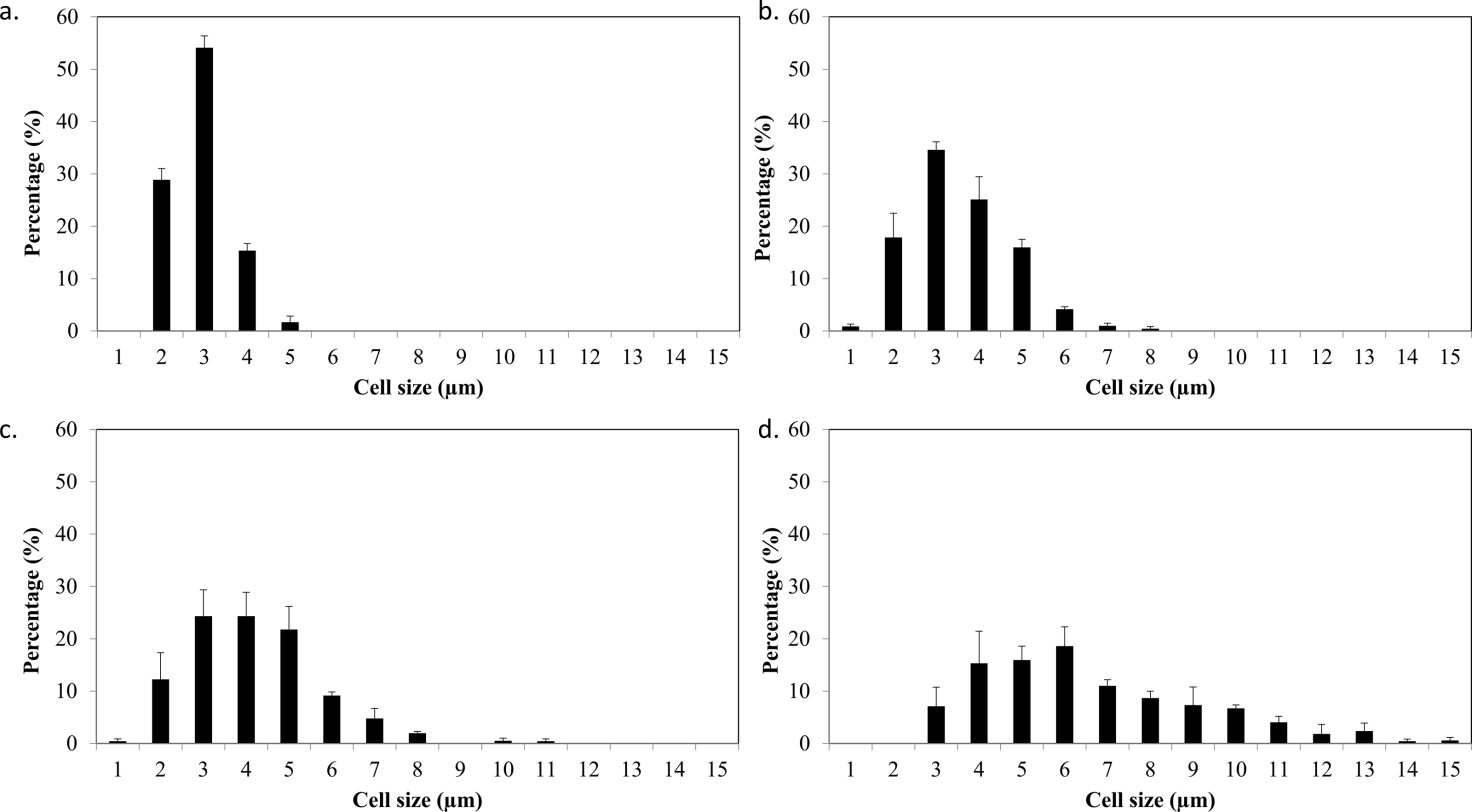
Cell size distribution of *C. sorokiniana* and *C. zofingiensis* in photoautotrophic and mixotrophic conditions. (A) *C. sorokiniana* in photoautotrophic condition, (B) *C. sorokiniana* in mixotrophic condition, (C) *C. zofingiensis* in photoautotrophic condition, and (D) *C. zofingiensis* in mixotrophic condition.

Based on the results obtained, the mixotrophic culture triggered morphological changes in the *Chlorella* cells. The most obvious changes were to the cell size, as both *Chlorella* species grew larger when cultured under mixotrophic condition (Figs. 2 and 3). The scanning electron micrographs clearly showed that the cell sizes increased due to the increased amounts of cell contents. Figure 4 shows the distribution of cell sizes under different culture conditions for *C. sorokiniana* and *C. zofingiensis*. On average, the size of *C. sorokiniana* under photoautotrophic condition was 2 to 4 µm. When a higher light intensity and glucose were introduced to the mixotrophic condition, the size of *C. sorokiniana* increased slightly to 3 to 5 µm and in some cases, even reached to 7 to 8 µm. Compared to *C. sorokiniana*, *C. zofingiensis* cells doubled in size when cultured under mixotrophic condition, from approximately 4 µm to 6 to 9 µm. Our results were similar to those of George et al. (2014), who studied the effects of light intensity on cell morphology and found that the cell shapes changed and the cell sizes increased in cultures grown under 150 µmol photons m^−1^ s^−1^. Several other researchers have studied the individual or combined effects of different environmental and nutritional conditions on cell morphology; those studies also reported that the sizes of microalgae cells increased when culture conditions were introduced (Latasa & Berdalet, 1994; Chokshi et al., 2015). Most of such studies found that the size of the cells increased 1- to 2-fold, regardless of the microalgae species.

**Figure 5:**
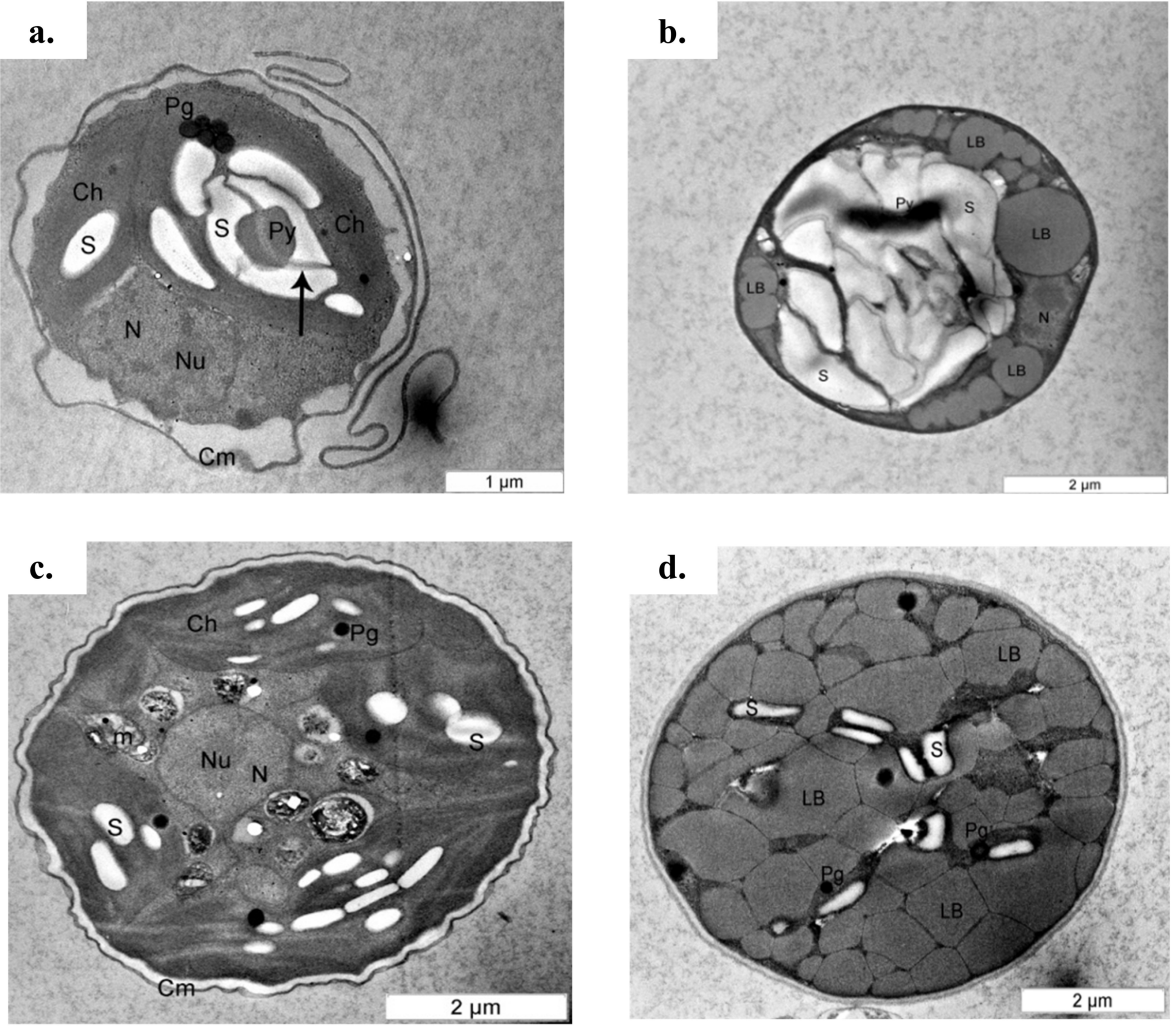
Transmission electron micrograph of *C. sorokiniana* and *C. zofingiensis*. (A) *C. sorokiniana* in photoautotrophic condition, (B) *C. sorokiniana* in mixotrophic condition, (C) *C. zofingiensis* in photoautotrophic condition, and (D) *C. zofingiensis* in mixotrophic condition. S, starch granule; N, nucleus, Nu, nucleolus; Ch, chloroplast; Py, pyrenoid; Pg, plastoglobule; Cm, cell membrane.

The cell content also changed under the mixotrophic condition in this study. This was evidenced by TEM ultrastructure evaluation (Fig. 5). A previous study proposed that mature *Chlorella* cells contain multiple parietal chloroplasts, whereas younger cells contain a single nucleus and a single parietal chloroplast (Fučíková & Lewis, 2012). The presence of multiple chloroplasts in *Chlorella* species assists with the construction of larger cell sizes due to the simultaneous accumulation of glycolytic lipids in storage vesicles and photosynthetic carbon fixation (Rosenberg et al., 2014), and multiple chloroplasts were observed in both *C. sorokiniana* and *C. zofingiensis* in this study. However, electron micrographs also showed that though *C. sorokiniana* had a lower number of chloroplasts, these chloroplasts were larger than those of *C. zofingiensis* under photoautotrophic condition. Wan et al. (2011) reported that *C. sorokiniana* expresses acetyl-coA carboxylase at higher levels in the cytosol than in the chloroplast under mixotrophic culture condition, suggesting that this species is less dependent on photosynthetically fixed carbon for lipid synthesis. Thus, fewer chloroplasts are needed to achieve sufficient amount of lipid accumulation. Future studies should compare the expression levels of acetyl-coA carboxylase in the cytosol and the chloroplasts in *C. zofingiensis* to further support this idea. In addition to the chloroplasts, *C. sorokiniana* also had more pyrenoid starch and larger plastoglobules than *C. zofingiensis*. When the cells were cultured under mixotrophic condition, the lipid bodies and starch formation, which accumulated in the middle of the cells and were surrounded by a lipid body, were predominant in both *C. sorokiniana* and *C. zofingiensis*. Similar findings have also been reported in other strains of microalgae (Siaut et al., 2011; Yao et al., 2012; George et al., 2014). However, previous studies have shown that the biosynthesis of starch and lipid bodies are not necessarily proportional and that their accumulation is strain-dependent and variable depending on the medium and culture conditions (Siaut et al., 2011; Takeshita et al., 2014). Based on the observations of the present study, we confirmed that moderate light intensity was sufficient to trigger changes under mixotrophic culture condition. Moreover, this change did not necessarily require the use of higher light intensities that were previously reported in the literature (Del Campo et al., 2000; Cazzaniga et al., 2014).

### Pigment contents

The pigment contents of *C. sorokiniana* and *C. zofingiensis* under photoautotrophic and mixotrophic conditions are shown in Table 1. Based on the results presented in Table 1, chlorophyll a was the most comment pigment found in both microalgae under photoautotrophic condition, followed by chlorophyll b and total carotene. This result is explained by the fact that chlorophyll a is the principal pigment in microalgae, whereas chlorophyll b is an accessory pigment that collects energy, which is then passed on to chlorophyll a. Chlorophyll a and b are widely studied pigments commonly found in other *Chlorella* species (Ip & Chen, 2005). In the present study, *C. sorokiniana* had a higher chlorophyll a content (17.929 µg/mg dry weight of sample) compared with *C. zofingiensis* (15.690 µg/mg dry weight of sample). Chlorophyll a content in *C. sorokiniana* was also higher than those reported for other *Chlorella* species (Da Silva Gorgônio, Aranda & Couri, 2013; Goiris et al., 2015; Safafar et al., 2015). When the cells were cultured under the mixotrophic condition, the chlorophyll a and b contents dropped by 80% compared with those of cells cultured under photoautotrophic condition. These reductions in the chlorophyll contents under the mixotrophic condition were correlated with relieve of photoinhibition under high light intensity. This finding was similar to those of several previous studies (Ip, Wong & Chen, 2004; Liu et al., 2009; Abreu et al., 2012).

**Table 1:** Pigments present in the microalgae *C. sorokiniana* and *C. zofingiensis* under photoautotrophic and mixotrophic conditions.

Condition	Sample	Chlorophyll a (µg/mg dws)	Chlorophyll b (µg/mg dws)	Total carotene (µg/mg dws)
Photoautotrophic	*C. sorokiniana*	17.929 ± 0.027	6.436 ± 0.040	3.882 ± 0.017
	*C. zofingiensis*	15.690 ± 0.003	7.311 ± 0.054	4.005 ± 0.046
Mixotrophic	*C. sorokiniana*	2.593 ± 0.005	1.127 ± 0.013	5.256 ± 0.217
	*C. zofingiensis*	2.598 ± 0.025	1.431 ± 0.051	5.805 ± 0.012

**Notes.**

Data are mean value of three replicates ± SD. DWS: dry weight of sample.

The content of total carotenoids in both microalgae species increased by 30–40%, which was comparable with increase of the carotenoid content reported in the literature (Matsukawa et al., 2000; Ip, Wong & Chen, 2004; Goiris et al., 2015; Safafar et al., 2015). As shown in Table 1, the amount of total carotenoid content per dry weight of the sample was almost the same under both conditions; this result is in contrast to those for chlorophylls a and b. Thus, we postulated that compared with the photoautotrophic culture condition, decreased amount of primary carotenoids and excessive amounts of secondary carotenoids were produced under conditions with higher light intensities and the addition of glucose in mixotrophic condition. Mulders et al. (2014) have stated that prolonged growth under limited light condition resulted in extremely low or absent concentrations of secondary carotenoids, whereas primary carotenoids were generally present at maximal concentrations. By contrast, other studies have shown that under mixotrophic culture conditions, primary carotenoids are generally degraded (although certain green algae produce excessive amounts of secondary carotenoids) (Leya et al., 2009; Mulders et al., 2014). The changes in carotenoid content were also shown in this study by the discolouration of *C. sorokiniana* from greenish to a pale green or yellowish green and of *C. zofingiensis* from green to red or orange (Fig. 2). These results were similar to those reported in previous studies, in which the primary carotenoids decreased with the onset of the red phase (Leya et al., 2009; Mulders et al., 2014). Furthermore, in microalgae grown under mixotrophic condition, the production of secondary carotenoids is always observed because carotenoids serve as photoprotective compounds that prevent photooxidative damage to photoautotrophic cells (Solovchenko et al., 2008). Thus, higher carotenoid content indicates higher cell survival rates. Compared with synthetic carotenoids, natural carotenoids from microalgae offer a greater commercial advantage with high bioavailability and lower toxicity compared to synthetic carotenoids (Priyadarshani & Rath, 2012).

Based upon previous studies, the type of carotenoid commonly produced by *C. sorokiniana* is lutein (Matsukawa et al., 2000; Cordero et al., 2011) whereas *C. zofingiensis* produces astaxanthin (Liu et al., 2014). Under mixotrophic culture condition, *C. sorokiniana* and *C. zofingiensis* exhibited yellow and red colours (Fig. 2) that corresponded to the colour of lutein and astaxanthin, respectively (Gupta et al., 2007). When the microalgal culture was exposed to a higher light intensity, secondary carotenoids such as astaxanthin were produced to filter the higher light intensity, preventing photons from being absorbed by the photosynthesis reaction centre (Vonshak & Torzillo, 2004). A previous study by Vonshak & Torzillo (2004) has shown that these carotenoids usually accumulate inside and/or outside the chloroplast. Thus, in the present study, the colours of the cells changed according to which pigments were produced (Fig. 2). In addition, previous research has shown that under mixotrophic conditions, some carotenoids, such as β-carotene, accumulate in globules outside the chloroplast and protect the reaction centre from excessive excitation by absorbing the light and reducing the amount of energy transferred to the reaction centre (Vonshak & Torzillo, 2004). This phenomenon was observed in the TEM micrographs where the globular structures representing the accumulation of lipids and/or lipid-soluble substances such as carotenoids was seen (Fig. 5).

**Figure 6:**
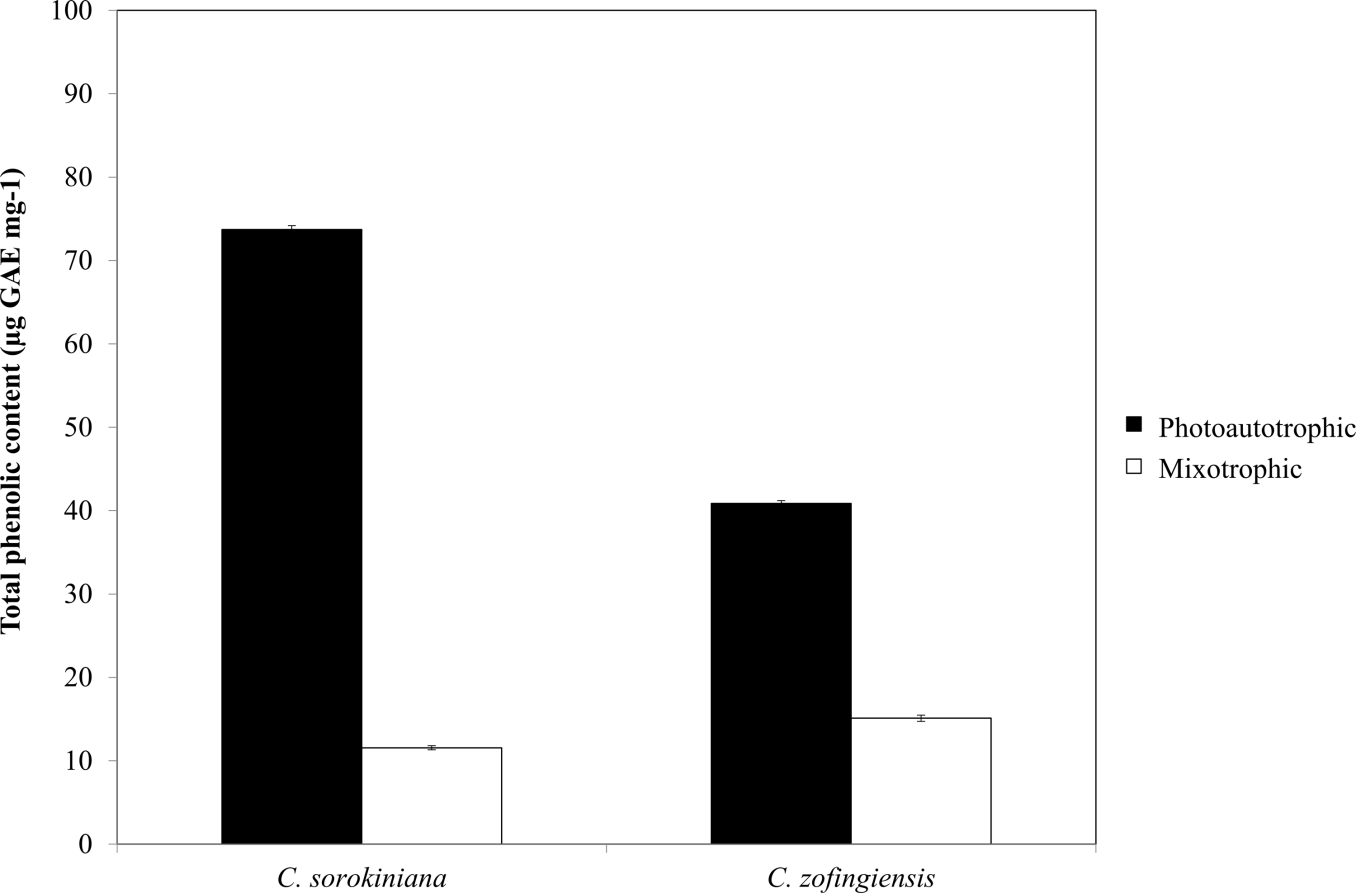
Total phenolic content of *C. sorokiniana* and *C. zofingiensis* under photoautotrophic and mixotrophic conditions.

### Total phenolic content

The phenolic compounds that are commonly found in plants and microalgae have been reported to have a wide range of biological activities, including antioxidant properties. Being among the most important antioxidants, phenolic compounds have the ability to donate a hydrogen atom or an electron to form stable radical intermediates. Based on a previous study, the Folin-Ciocalteu method was used to study the total phenolic content of microalgae (Ndhlala, Moyo & Staden, 2010). The total phenolic contents of ethanol extracts of both *Chlorella* species under photoautotrophic and mixotrophic conditions are presented in Fig. 6. In both species, total phenolic content was higher under the photoautotrophic condition than under the mixotrophic condition for both species, at 73.7 µg GAE mg^−1^ for *C. sorokiniana* and 40.8 µg GAE mg^−1^ for *C. zofingiensis*. When the cells were cultured under the mixotrophic condition, the total phenolic content was reduced by up to 84.4% in *C. sorokiniana* and 63% in *C. zofingiensis*, resulting in total phenolic contents of 11.56 µg GAE mg^−1^ and 15.10 µg GAE mg^−1^, respectively. This contradicts a previous report, which claimed that mixotrophic condition was best to produce higher phenolic contents in *Chlorella* species (Shetty & Sibi, 2015). Although the phenolic content was reduced under the mixotrophic condition, the phenolic content was higher than that reported in the literature for other microalgae (Goiris et al., 2012; Hemalatha et al., 2013; Saranya et al., 2014; Safafar et al., 2015). For example, Saranya et al. (2014) compared the biochemical contents of different microalgae and observed that *Isochrysis* spp. had the highest phenolic content with only 4.57 mg GAE g^−1^. In another study, Ali et al. (2014) screened different microalgae for their carotenoids and phenolic contents and found a high phenolic content (39.1 mg GAE g^−1^) in *Chlorella* spp. Goiris et al. (2015) reported that though the production of carotenoids and phenolic contents were reduced under nutrient-limited conditions, the production of ascorbic acid and tocopherols both increased. Thus, in our case, harvesting at the end of the stationary phase might contribute to the reduction of phenolic contents. Moreover, Goiris et al. (2012) found that the antioxidant content of different microalgae varied greatly between species and was dependent on extraction strategy.

**Figure 7:**
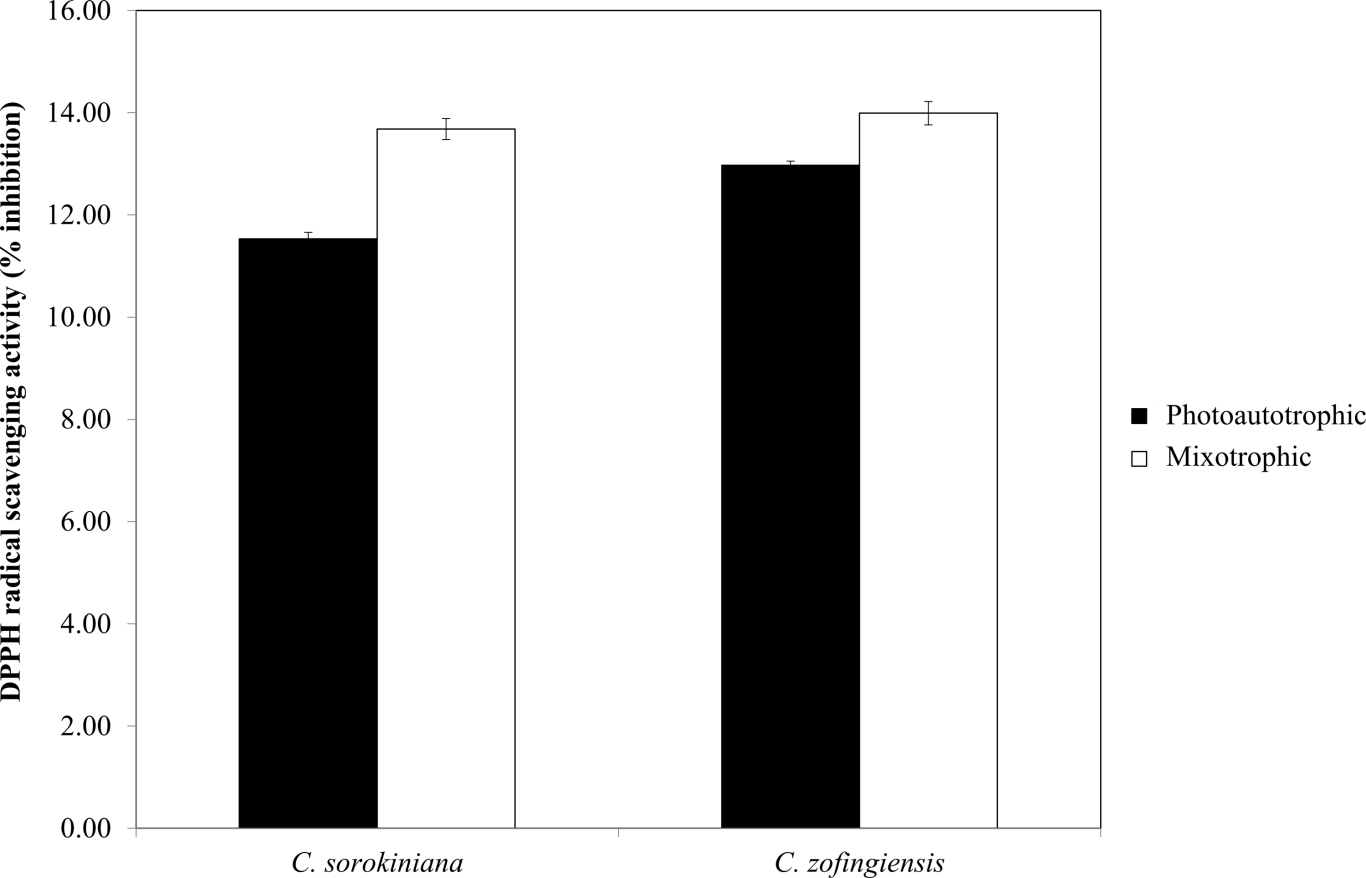
Percentage of DPPH radical scavenging activity by *C. sorokiniana* and *C. zofingiensis* under photoautotrophic and mixotrophic conditions.

**Figure 8:**
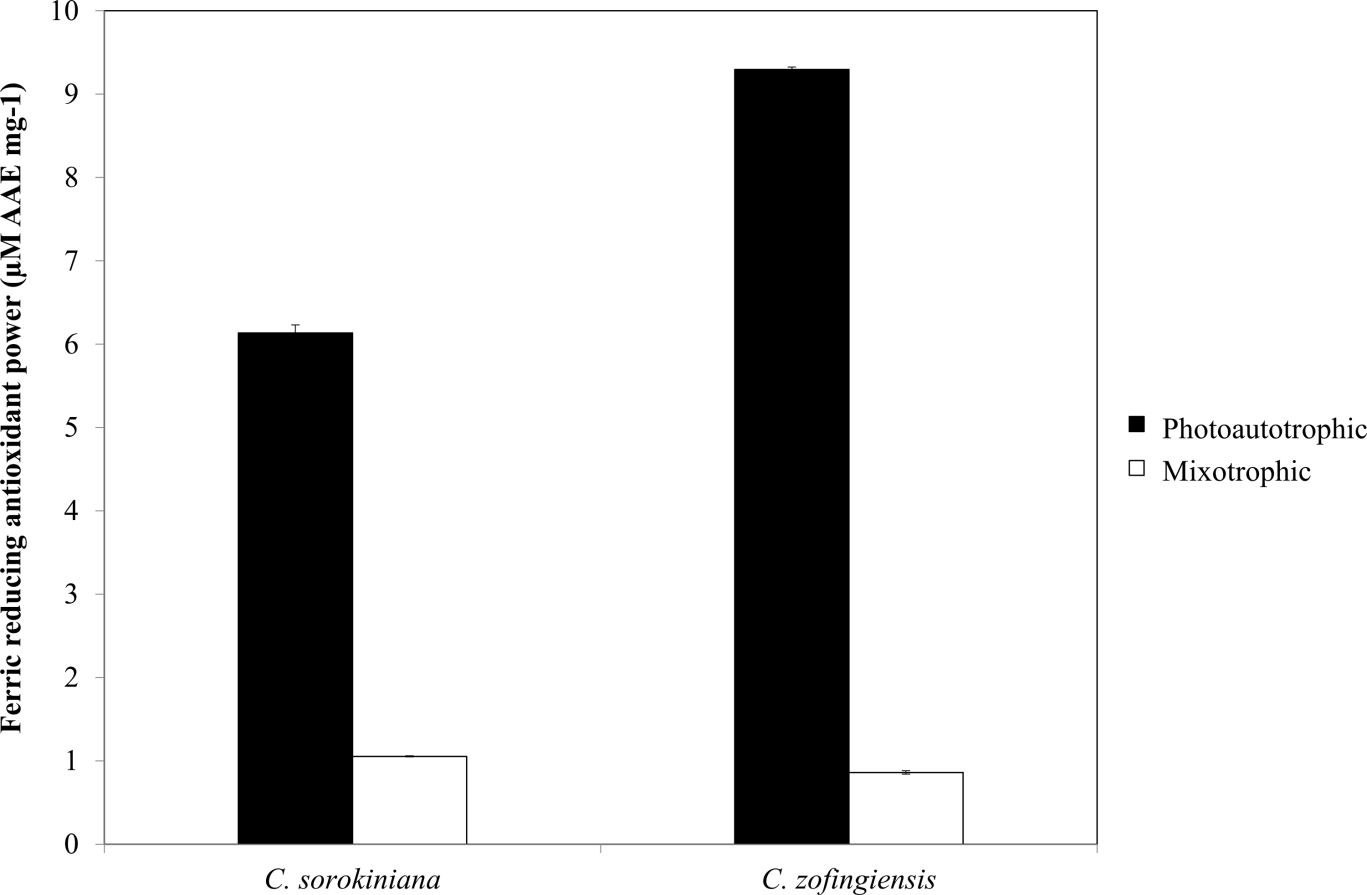
Ferric reducing antioxidant power of *C. sorokiniana* and *C. zofingiensis* under photoautotrophic and mixotrophic conditions.

### Antioxidant activity

The DPPH radical scavenging activities (%) of *C. sorokiniana* and *C. zofingiensis* under photoautotrophic and mixotrophic conditions are presented in Fig. 7. Extracts from both strains of microalgae cultured under photoautotrophic and mixotrophic conditions showed the ability to scavenge DPPH to the same degree. Compared with *C. sorokiniana*, which showed 11.5% and 13.7% scavenging activities under photoautotrophic and mixotrophic conditions, respectively, *C. zofingiensis* showed slightly stronger scavenging activities of 13% and 14%, respectively. Generally, the percentage of scavenging activity measured in this study was within the range obtained for different microalgae (9 to 35%), as reported by previous studies (Hemalatha et al., 2013; Saranya et al., 2014; Safafar et al., 2015). For example, Hemalatha et al. (2013) investigated the antioxidant properties of different microalgae, such as *Navicula clavata*, *Chlorella marina* and *Dunaliella salina*, and reported DPPH scavenging activities ranging from 9% to 24%. Meanwhile, Saranya et al. (2014) and Safafar et al. (2015) showed that the methanolic extracts of *Isochrysis galbana* and *Chlorella sorokiniana* had the highest DPPH scavenging activities, with each approximately 34%.

As indicated by FRAP assay, the reducing power of both *Chlorella* species was higher under photoautotrophic condition than under mixotrophic condition (Fig. 8). Specifically, under photoautotrophic condition, *C. zofingiensis* showed a higher reducing power activity than *C. sorokiniana* with FRAP values of 9.29 ± 0.029 µM AAE mg^−1^ and 6.13 ± 0.097 µM AAE mg^−1^, respectively. On the other hand, under mixotrophic condition, both species showed FRAP values of approximately 1 µM AAE mg^−1^. FRAP detects antioxidants that act through single electron transfer, but cannot detect compounds that act as radical quenchers via hydrogen atom transfer (Prior, Wu & Schaich, 2005) a phenomenon that is mainly carried out by phenols with the ability to transfer single electrons. Based on the FRAP results, the antioxidants activities tended to be proportional to the phenolic content. Thus, the FRAP assay results represent the antioxidant activities of the phenolic compounds. Although the total phenolic content of *C. zofingiensis* was lower than that of *C. sorokiniana*, the antioxidant activity detected by the FRAP assay was high. This result might have been due to the presence of different types of antioxidant compounds that are similar to phenols, such as flavonoids, tocopherols and vitamin C (Ndhlala, Moyo & Staden, 2010). More interestingly, the reducing activities of these microalgal extracts were higher compared to those reported in the literature, as most of the previously evaluated microalgae, such as *Navicula clavata*, *Chlorella marina*, *Dunaliella salina*, *Chaetoceros calcitrans*, *Chlorella salina* and *Isochrysis galbana*, have reducing activities below 1 mg AAE g^−1^ (Uma, Sivasubramanian & Devaraj, 2011; Hemalatha et al., 2013; Saranya et al., 2014).

The results of the DPPH and FRAP assays showed that the former detected more antioxidant activity in the mixotrophic samples than the latter. This result may have several explanations. First, DPPH has limitations when assessing carotenoid samples, as some carotenoids molecules have absorbance wavelengths of 517 nm that overlap with the DPPH signal in absorbance spectra (Arnao, 2000; Pérez-Jiménez et al., 2008). Second, in the DPPH assay, steric accessibility is the major determinant of the reaction mechanisms, thus, small molecules such as carotenoids have higher apparent antioxidant capacities due to their better access to the DPPH radical site (Prior, Wu & Schaich, 2005). Third, some protein and thiol antioxidants, such as glutathione, cannot be measured by the FRAP assay (Ndhlala, Moyo & Staden, 2010). This steric accessibility might also affect the antioxidant activity detected by the FRAP assay under mixotrophic condition, in which glutathione is produced in greater quantities (Cheng & He, 2014).

Comparing the results obtained for both *Chlorella* species, *C. zofingiensis* showed a slightly higher antioxidant activity than *C. sorokiniana*. Although the total phenolic content of *C. sorokiniana* was higher than that of *C. zofingiensis*, the FRAP result for *C. zofingiensis* was higher, indicating that *C. zofingiensis* possessed greater antioxidant activity than *C. sorokiniana*. This result suggests that different species of microalgae respond to mixotrophic condition in different ways, leading to the production of different classes of antioxidants. In addition, different classes of antioxidants respond in different ways under different culture conditions. For example, the antioxidant activities of carotenoids and phenolic compounds were not the same under photoautotrophic and mixotrophic conditions. Specifically, the antioxidant activity of phenolic compounds was high under photoautotrophic condition, but low under mixotrophic condition, whereas the total carotene antioxidant activity was high under both conditions.

## Conclusions

This work demonstrated the different responses of *Chlorella sorokiniana* and *Chlorella zofingiensis* to different culture conditions. The antioxidant activities measured by the DPPH and FRAP assays represented the carotenoid and phenolic contents of the microalgae cultured under photoautotrophic and mixotrophic conditions. Phenolic compounds were produced at higher levels under the photoautotrophic condition, whereas carotenoids were produced to the same degree under both photoautotrophic and mixotrophic conditions. This indicates that different metabolites were produced under different culture conditions. In addition, the levels of antioxidants in both *Chlorella* species also differed with regards to the phenolic and carotenoid contents. Hence, future studies profiling the carotenoids and polyphenols using HPLC and LC-MS are of high priority, as these works will improve the understanding of the detailed changes of these important metabolites under the mixotrophic condition. Thus, the manipulation of the conditions used to culture a specific microalgal species is very important for the production of the desired metabolites. Production under photoautotrophic or mixotrophic conditions would affect the commercial application of *Chlorella sorokiniana* and *Chlorella zofingiensis*, especially in downstream processes, as different products require different processing methods and system setups.

##  Supplemental Information

10.7717/peerj.3473/supp-1 Data S1 Raw data

## Additional Information and Declarations

### Data Availability

The following information was supplied regarding data availability: The raw data has been supplied as Data S1.

## References

[ref-1] Abreu AP, Fernandes B, Vicente AA, Teixeira J, Dragone G (2012). Mixotrophic cultivation of *Chlorella vulgaris* using industrial dairy waste as organic carbon source. Bioresource Technology.

[ref-2] Ali HEA, Shanab SMM, Shalaby EAA, Eldmerdash U, Abdullah MA (2014). Screening of microalgae for antioxidant activities, carotenoids and phenolic contents. Applied Mechanics and Materials.

[ref-3] Arnao MB (2000). Some methodological problems in the determination of antioxidant activity using chromogen radicals: a practical case. Trends in Food Science & Technology.

[ref-4] Brányiková I, Maršálková B, Doucha J, Brányik T, Bišová K, Zachleder V, Vítová M (2011). Microalgae—novel highly efficient starch producers. Biotechnology and Bioengineering.

[ref-5] Cardozo KH, Guaratini T, Barros MP, Falcao VR, Tonon AP, Lopes NP, Campos S, Torres MA, Souza AO, Colepicolo P, Pinto E (2007). Metabolites from algae with economical impact. Comparative Biochemistry and Physiology. Toxicology & Pharmacology: CBP.

[ref-6] Cazzaniga S, Dall’Osto L, Szaub J, Scibilia L, Ballottari M, Purton S, Bassi R (2014). Domestication of the green alga *Chlorella sorokiniana*: reduction of antenna size improves light-use efficiency in a photobioreactor. Biotechnology for Biofuel.

[ref-7] Chacón-Lee TL, González-Mariño GE (2010). Microalgae for “healthy” foods—possibilities and challenges. Comprehensive Reviews in Food Science and Food Safety.

[ref-8] Cheng D, He Q (2014). Assessment of environmental stresses for enhanced microalgal biofuel production—an overview. Frontiers in Energy Research.

[ref-9] Chokshi K, Pancha I, Trivedi K, George B, Maurya R, Ghosh A, Mishra S (2015). Biofuel potential of the newly isolated microalgae *Acutodesmus dimorphus* under temperature induced oxidative stress conditions. Bioresource Technology.

[ref-10] Cordero BF, Obraztsova I, Couso I, Leon R, Vargas MA, Rodriguez H (2011). Enhancement of lutein production in *Chlorella sorokiniana* (Chorophyta) by improvement of culture conditions and random mutagenesis. Marine Drugs.

[ref-11] Cox S, Abu-Ghannam N, Gupta S (2010). An assessment of the antioxidant and antimicrobial activity of six species of edible Irish seaweeds. International Food Research Journal.

[ref-12] Da Silva Gorgônio CM, Aranda DAG, Couri S (2013). Morphological and chemical aspects of *Chlorella pyrenoidosa*, *Dunaliella tertiolecta, Isochrysis galbana* and *Tetraselmis gracilis* microalgae. Natural Science.

[ref-13] Darko E, Heydarizadeh P, Schoefs B, Sabzalian MR (2014). Photosynthesis under artificial light: the shift in primary and secondary metabolism. Philosophical Transactions of Royal Society B.

[ref-14] De Morais MG, Vaz BDS, De Morais EG, Costa JAV (2015). Biologically active metabolites snthesized by microalgae. BioMed Research International.

[ref-15] Del Campo JA, Moreno J, Rodríguez H, Vargas MA, Rivas J, Guerrero MG (2000). Carotenoid content of chlorophycean microalgae: factors determining lutein accumulation in *Muriellopsis* sp . (Chlorophyta). Journal of Biotechnology.

[ref-16] Del Campo JA, Rodriguez H, Moreno J, Vargas MA, Rivas J, Guerrero MG (2004). Accumulation of astaxanthin and lutein in *Chlorella zofingiensis* (Chlorophyta). Applied Microbiology Biotechnology.

[ref-17] Del Campo JA, García-González M, Guerrero MG (2007). Outdoor cultivation of microalgae for carotenoid production: current state and perspectives. Applied Microbiology and Biotechnology.

[ref-18] Fučíková K, Lewis LA (2012). Intersection of *Chlorella, Muriella and Bracteacoccus*: resurrecting the genus *Chromochloris* KOL et CHODAT (Chlorophyceae, Chlorophyta). Journal of the Czech Phycological Society.

[ref-19] George B, Pancha I, Desai C, Chokshi K, Paliwal C, Ghosh T, Mishra S (2014). Effects of different media composition, light intensity and photoperiod on morphology and physiology of freshwater microalgae *Ankistrodesmus falcatus*—a potential strain for biofuel production. Bioresource Technology.

[ref-20] Goiris K, Muylaert K, Fraeye I, Foubert I, De Brabanter J, De Cooman L (2012). Antioxidant potential of microalgae in relation to their phenolic and carotenoid content. Journal of Applied Phycology.

[ref-21] Goiris K, Van Colen W, Wilches I, León-Tamariz F, De Cooman L, Muylaert K (2015). Impact of nutrient stress on antioxidant production in three species of microalgae. Algal Research.

[ref-22] Grünewald K, Hirschberg J, Hagen C (2001). Ketocarotenoid biosynthesis outside of plastids in the unicellular green alga *Haematococcus pluvialis*. Journal of Biological Chemistry.

[ref-23] Gupta S, Jha A, Pal A, Venkateshwarlu G (2007). Use of natural carotenoids for pigmentation in fishes. Natural Product Radiance.

[ref-24] Hajimahmoodi M, Faramarzi MA, Mohammadi N, Soltani N, Oveisi MR, Nafissi-Varcheh N (2010). Evaluation of antioxidant properties and total phenolic contents of some strains of microalgae. Journal of Applied Phycology.

[ref-25] Hemalatha A, Girija K, Parthiban C, Saranya C, Anantharaman P (2013). Antioxidant properties and total phenolic content of a marine diatom, green microalgae. Advances in Applied Science Research.

[ref-26] Hu Q, Richmond A (2004). Environmental effects on cell composition. Handbook of microalgal culture: biotechnology and applied phycology.

[ref-27] Ip P-F, Chen F (2005). Production of astaxanthin by the green microalga *Chlorella zofingiensis* in the dark. Process Biochemistry.

[ref-28] Ip P-F, Wong K-H, Chen F (2004). Enhanced production of astaxanthin by the green microalga *Chlorella zofingiensis* in mixotrophic culture. Process Biochemistry.

[ref-29] Iwamoto H (2004). Chapter 11. Industrial production of microalgal cell-mass and secondary products—major industrial species : Chlorella.

[ref-30] Jorquera Q, Kiperstok A, Sales EA, Embirucu M, Ghirardi ML (2010). Comparative energy life-cycle analyses of microalgal biomass production in open ponds and photobioreactors. Bioresource Technology.

[ref-31] Juneja A, Ceballos RM, Murthy GS (2013). Effects of environmental factors and nutrient availability on the biochemical composition of algae for biofuels production: a review. Energies.

[ref-32] Kumar K, Dasgupta CN, Das D (2014). Cell growth kinetics of *Chlorella sorokiniana* and nutritional values of its biomass. Bioresource Technology.

[ref-33] Latasa M, Berdalet E (1994). Effect of nitrogen or phosphorus starvation on pigment composition of cultured *Heterocapsa* sp. Journal of Plankton Research.

[ref-34] Leopoldini M, Russo N, Toscano M (2011). The molecular basis of working mechanism of natural polyphenolic antioxidants. Food Chemistry.

[ref-35] Leya T, Rahn A, Lütz C, Remias D (2009). Response of arctic snow and permafrost algae to high light and nitrogen stress by changes in pigment composition and applied aspects for biotechnology. FEMS Microbiology Ecology.

[ref-36] Liang Y, Sarkany N, Cui Y (2009). Biomass and lipid productivities of *Chlorella vulgaris* under autotrophic, heterotrophic and mixotrophic growth conditions. Biotechnology Letters.

[ref-37] Lichtenthaler HK, Buschmann C (2001). Chlorophylls and carotenoids: measurement and characterization by UV—VIS spectroscopy. Current Protocols in Food Analytical Chemistry.

[ref-38] Liu X, Duan S, Li A, Xu N, Cai Z, Hu Z (2009). Effects of organic carbon sources on growth, photosynthesis, and respiration of *Phaeodactylum tricornutum*. Journal of Applied Phycology.

[ref-39] Liu J, Sun Z, Gerken H, Liu Z, Jiang Y, Chen F (2014). *Chlorella zofingiensis* as an alternative microalgal producer of astaxanthin: biology and industrial potential. Marine Drugs.

[ref-40] Lodish H, Berk A, Zipursky SL, Matsudaira P, Baltimore D, Darnell J (2000). Photosynthetic stages and light-absorbing pigments. Molecular cell biology.

[ref-41] Masojídek J, Koblízek M, Torzillo G, Richmond A (2004). Photosynthesis in microalgae. Handbook of microalgal culture: biotechnology and applied phycology.

[ref-42] Matsukawa R, Hotta M, Masuda Y, Chihara M, Karube I (2000). Antioxidants from carbon dioxide fixing *Chlorella sorokiniana*. Journal of Applied Phycology.

[ref-43] Mimouni V, Ulmann L, Pasquet V, Mathieu M, Picot L, Bougaran G, Morant-Manceau A, Schoefs B (2012). The potential of microalgae for the production of bioactive molecules of pharmaceutical interest. Current Pharmaceutical Biotechnology.

[ref-44] Mostafa SS, Dhal NK (2012). Microalgal biotechnology: prospects and applications. Plant science.

[ref-45] Mulders KJM, Weesepoel Y, Bodenes P, Lamers PP, Vincken JP, Martens DE, Gruppen H, Wijffels RH (2014). Nitrogen-depleted *Chlorella zofingiensis* produces astaxanthin, ketolutein and their fatty acid esters: a carotenoid metabolism study. Journal of Applied Phycology.

[ref-46] Ndhlala AR, Moyo M, Van Staden J (2010). Natural antioxidants: fascinating or mythical biomolecules?. Molecules.

[ref-47] Pérez-Jiménez J, Arranz S, Tabernero M, Díaz-Rubio ME, Serrano J, Goñi I, Saura-Calixto F (2008). Updated methodology to determine antioxidant capacity in plant foods, oils and beverages: extraction, measurement and expression of results. Food Research International.

[ref-48] Prior RL, Wu X, Schaich K (2005). Standardized methods for the determination of antioxidant capacity and phenolics in foods and dietary supplements. Journal of Agricultural and Food Chemistry.

[ref-49] Priyadarshani I, Rath B (2012). Commercial and industrial applications of micro algae—a review. Journal of Algal Biomass Utilization.

[ref-50] Rodriguez-Garcia I, Guil-Guerrero JL (2008). Evaluation of the antioxidant activity of three microalgal species for use as dietary supplements and in the preservation of foods. Food Chemistry.

[ref-51] Rosenberg JN, Kobayashi N, Barnes A, Noel EA, Betenbaugh MJ, Oyler GA (2014). Comparative analyses of three *Chlorella* species in response to light and sugar reveal distinctive lipid accumulation patterns in the microalga *C. sorokiniana*. PLOS ONE.

[ref-52] Safafar H, Van Wagenen J, Møller P, Jacobsen C (2015). Carotenoids, phenolic compounds and tocopherols contribute to the antioxidative properties of some microalgae species grown on industrial wastewater. Marine Drugs.

[ref-53] Saranya C, Hemalatha A, Parthiban C, Anantharaman P (2014). Evaluation of antioxidant properties, total phenolic and carotenoid content of *Chaetoceros calcitrans*, *Chlorella salina* and *Isochrysis galbana*. International Journal of Current Microbiology and Applied Science.

[ref-54] Sawant SS, Joshi AA, Bhagwat A, Kelkar-Mane V (2014). Tapping the antioxidant potential of a novel isolate-*Chlorella Emersonii*. World Journal of Pharmaceutical Research.

[ref-55] Shetty V, Sibi G (2015). Relationship between total phenolics content and antioxidant activities of microalgae under autotrophic, heterotrophic and mixotrophic growth. Journal of Food Resource Science.

[ref-56] Siaut M, Cuiné S, Cagnon C, Fessler B, Nguyen M, Carrier P, Beyly A, Beisson F, Triantaphylidès C, Li-Beisson Y (2011). Oil accumulation in the model green alga *Chlamydomonas reinhardtii*: characterization, variability between common laboratory strains and relationship with starch reserves. BMC Biotechnology.

[ref-57] Skjanes K, Rebours C, Lindblad P (2013). Potential for green microalgae to produce hydrogen, pharmaceuticals and other high value products in a combined process. Critical Reviews in Biotechnology.

[ref-58] Solovchenko A, Khozin-Goldberg I, Didi-Cohen S, Cohen Z, Merzlyak M (2008). Effects of light intensity and nitrogen starvation on growth, total fatty acids and arachidonic acid in the green microalga *Parietochloris incisa*. Journal of Applied Phycology.

[ref-59] Spolaore P, Joannis-Cassan C, Duran E, Isambert A (2006). Commercial applications of microalgae. Journal of Bioscience and Bioengineering.

[ref-60] Taga MS, Miller EE, Pratt DE (1984). Chia seeds as a source of natural lipid antioxidants. Journal of the American Oil Chemists’ Society.

[ref-61] Takaichi S (2011). Carotenoids in algae: distributions, biosyntheses and functions. Marine Drugs.

[ref-62] Takeshita T, Ota S, Yamazaki T, Hirata A, Zachleder V, Kawano S (2014). Starch and lipid accumulation in eight strains of six *Chlorella* species under comparatively high light intensity and aeration culture conditions. Bioresource Technology.

[ref-63] Uma R, Sivasubramanian V, Devaraj SN (2011). Evaluation of *in vitro* antioxidant activities and antiproliferative activity of green microalgae, *Desmococcus olivaceous* and *Chlorococcum humicola*. Journal of Algal Biomass Utilization.

[ref-64] Vachali P, Bhosale P, Bernstein PS, Barredo J-L (2012). Microbial carotenoids. Microbial carotenoids from fungi.

[ref-65] Vonshak A, Torzillo G, Richmond A (2004). Environmental stress physiology. Handbook of microalgal culture: biotechnology and applied phycology.

[ref-66] Wan M, Li P, Xia J, Rosenberg JN, Oyler GA, Betenbaugh MJ, Nie Z, Qiu G (2011). The effect of mixotrophy on microalgal growth, lipid content, and expression levels of three pathway genes in *Chlorella sorokiniana*. Applied Microbiology and Biotechnology.

[ref-67] Wang Y, Chen T (2008). The biosynthetic pathway of carotenoids in the astaxanthin-producing green alga *Chlorella zofingiensis*. World Journal of Microbiology and Biotechnology.

[ref-68] Yang C, Hua Q, Shimizu K (2000). Energetics and carbon metabolism during growth of microalgal cells under photoautotrophic, mixotrophic and cyclic light-autotrophic/dark-heterotrophic conditions. Biochemical Engineering Journal.

[ref-69] Yao C, Ai J, Cao X, Xue S, Zhang W (2012). Enhancing starch production of a marine green microalga *Tetraselmis subcordiformis* through nutrient limitation. Bioresource Technology.

[ref-70] Zhu S, Huang W, Xu J, Wang Z, Xu J, Yuan Z (2014). Metabolic changes of starch and lipid triggered by nitrogen starvation in the microalga *Chlorella zofingiensis*. Bioresource Technology.

