# Supplementary material for: A comparison of the morphological and biochemical characteristics of Chlorella sorokiniana and Chlorella zofingiensis cultured under photoautotrophic and mixotrophic conditions
Source: PeerJ. 2017 Sep 8;5:e3473. doi: 10.7717/peerj.3473 (PMC5592954; doi:10.7717/peerj.3473)
Supplement: Data S1 [file peerj-05-3473-s001.docx]

Raw data for Paper: **Comparison of morphological and biochemical characteristic of *Chlorella* *sorokiniana* and *Chlorella* *zofingiensis*** **under light stress.**

**Fig. 3** Cell size distribution of *C. sorokiniana* and *C. zofingiensis* in normal and stress induced conditions.

Note: The number of cells depends on the cells captured in one picture of light microscope

1) Normal *C. sorokiniana* (NS):

| No. of cells | Diameter of cells (µm) | | |
| --- | --- | --- | --- |
|  | Replicate 1 | Replicate 2 | Replicate 3 |
| 1 | 5.293 | 4.663 | 5.173 |
| 2 | 4.498 | 4.642 | 5.170 |
| 3 | 4.470 | 4.487 | 5.004 |
| 4 | 4.379 | 4.447 | 4.611 |
| 5 | 4.317 | 4.381 | 4.559 |
| 6 | 4.307 | 4.273 | 4.490 |
| 7 | 4.256 | 4.161 | 4.490 |
| 8 | 4.256 | 4.143 | 4.402 |
| 9 | 4.104 | 4.074 | 4.396 |
| 10 | 4.073 | 4.035 | 4.371 |
| 11 | 4.044 | 4.035 | 4.323 |
| 12 | 4.032 | 3.996 | 4.259 |
| 13 | 4.006 | 3.971 | 4.248 |
| 14 | 3.970 | 3.937 | 4.196 |
| 15 | 3.960 | 3.931 | 4.126 |
| 16 | 3.960 | 3.885 | 4.062 |
| 17 | 3.937 | 3.874 | 4.005 |
| 18 | 3.910 | 3.872 | 3.942 |
| 19 | 3.910 | 3.867 | 3.938 |
| 20 | 3.879 | 3.844 | 3.932 |
| 21 | 3.816 | 3.801 | 3.927 |
| 22 | 3.810 | 3.767 | 3.916 |
| 23 | 3.808 | 3.739 | 3.827 |
| 24 | 3.744 | 3.699 | 3.790 |
| 25 | 3.738 | 3.648 | 3.751 |
| 26 | 3.672 | 3.632 | 3.710 |
| 27 | 3.660 | 3.606 | 3.675 |
| 28 | 3.617 | 3.578 | 3.670 |
| 29 | 3.600 | 3.544 | 3.650 |
| 30 | 3.570 | 3.535 | 3.614 |
| 31 | 3.558 | 3.499 | 3.554 |
| 32 | 3.518 | 3.494 | 3.539 |
| 33 | 3.510 | 3.476 | 3.524 |
| 34 | 3.510 | 3.465 | 3.524 |
| 35 | 3.480 | 3.445 | 3.516 |
| 36 | 3.480 | 3.402 | 3.516 |
| 37 | 3.446 | 3.386 | 3.480 |
| 38 | 3.369 | 3.380 | 3.450 |
| 39 | 3.353 | 3.349 | 3.444 |
| 40 | 3.308 | 3.345 | 3.421 |
| 41 | 3.298 | 3.342 | 3.406 |
| 42 | 3.286 | 3.314 | 3.377 |
| 43 | 3.280 | 3.294 | 3.293 |
| 44 | 3.276 | 3.294 | 3.280 |
| 45 | 3.273 | 3.276 | 3.276 |
| 46 | 3.267 | 3.248 | 3.264 |
| 47 | 3.252 | 3.124 | 3.244 |
| 48 | 3.220 | 3.120 | 3.244 |
| 49 | 3.135 | 3.103 | 3.231 |
| 50 | 3.090 | 3.093 | 3.231 |
| 51 | 3.086 | 3.093 | 3.224 |
| 52 | 3.076 | 3.055 | 3.181 |
| 53 | 3.069 | 3.054 | 3.158 |
| 54 | 3.055 | 3.052 | 3.154 |
| 55 | 3.004 | 3.035 | 3.117 |
| 56 | 2.985 | 3.034 | 3.088 |
| 57 | 2.982 | 3.031 | 3.080 |
| 58 | 2.982 | 3.024 | 3.021 |
| 59 | 2.968 | 3.021 | 2.975 |
| 60 | 2.925 | 2.992 | 2.964 |
| 61 | 2.913 | 2.986 | 2.953 |
| 62 | 2.910 | 2.979 | 2.949 |
| 63 | 2.862 | 2.968 | 2.934 |
| 64 | 2.862 | 2.942 | 2.898 |
| 65 | 2.858 | 2.932 | 2.898 |
| 66 | 2.809 | 2.928 | 2.872 |
| 67 | 2.787 | 2.909 | 2.872 |
| 68 | 2.772 | 2.903 | 2.858 |
| 69 | 2.769 | 2.892 | 2.858 |
| 70 | 2.769 | 2.862 | 2.799 |
| 71 | 2.635 | 2.849 | 2.785 |
| 72 | 2.583 | 2.841 | 2.688 |
| 73 | 2.530 | 2.766 | 2.641 |
| 74 | 2.525 | 2.766 | 2.495 |
| 75 | 2.477 | 2.764 | 2.440 |
| 76 | 2.445 | 2.746 | 2.359 |
| 77 | 2.427 | 2.746 | 2.232 |
| 78 | 2.422 | 2.688 | 2.051 |
| 79 | 2.404 | 2.665 |  |
| 80 | 2.328 | 2.541 |  |
| 81 | 2.070 | 2.499 |  |
| 82 | 2.048 | 2.372 |  |

|  | Percentage = (no. of cell in that size / total cells)*100 | | |  |  | error bar=std dev/ SQRT(n=number of reads) |
| --- | --- | --- | --- | --- | --- | --- |
| **cell size** | **NS1** | **NS2** | **NS3** | **average** | **std dev** | **std error** |
| 1 | 0 | 0 | 0 | 0.000 | 0.000 | 0.00 |
| 2 | 32.93 | 28.05 | 25.64 | 28.872 | 3.712 | 2.14 |
| 3 | 51.22 | 58.54 | 52.56 | 54.107 | 3.895 | 2.25 |
| 4 | 14.63 | 13.41 | 17.95 | 15.332 | 2.346 | 1.35 |
| 5 | 1.22 | 0.00 | 3.85 | 1.689 | 1.966 | 1.13 |

2) Stress *C. sorokiniana* (SS)

| No. of cells | Diameter of cells (µm) | | |
| --- | --- | --- | --- |
|  | Replicate 1 | Replicate 2 | Replicate 3 |
|  | SS1 | SS2 | SS3 |
| 1 | 7.524 | 8.419 | 6.552 |
| 2 | 6.399 | 7.426 | 6.420 |
| 3 | 6.381 | 6.861 | 6.386 |
| 4 | 5.612 | 6.255 | 5.895 |
| 5 | 5.532 | 6.138 | 5.866 |
| 6 | 5.487 | 6.080 | 5.817 |
| 7 | 5.461 | 5.750 | 5.775 |
| 8 | 5.427 | 5.627 | 5.760 |
| 9 | 5.393 | 5.485 | 5.713 |
| 10 | 5.380 | 5.462 | 5.647 |
| 11 | 5.321 | 5.376 | 5.563 |
| 12 | 5.271 | 5.297 | 5.551 |
| 13 | 4.992 | 5.271 | 5.522 |
| 14 | 4.830 | 5.165 | 5.510 |
| 15 | 4.815 | 5.147 | 5.373 |
| 16 | 4.663 | 5.110 | 5.309 |
| 17 | 4.525 | 5.031 | 5.222 |
| 18 | 4.525 | 4.912 | 4.954 |
| 19 | 4.460 | 4.899 | 4.946 |
| 20 | 4.337 | 4.899 | 4.939 |
| 21 | 4.286 | 4.816 | 4.922 |
| 22 | 4.204 | 4.798 | 4.917 |
| 23 | 4.168 | 4.691 | 4.772 |
| 24 | 4.114 | 4.691 | 4.741 |
| 25 | 3.937 | 4.582 | 4.727 |
| 26 | 3.922 | 4.582 | 4.669 |
| 27 | 3.867 | 4.479 | 4.567 |
| 28 | 3.861 | 4.359 | 4.460 |
| 29 | 3.851 | 4.339 | 4.366 |
| 30 | 3.803 | 4.321 | 4.321 |
| 31 | 3.801 | 4.307 | 4.320 |
| 32 | 3.769 | 4.236 | 4.219 |
| 33 | 3.641 | 4.163 | 4.204 |
| 34 | 3.594 | 4.095 | 4.197 |
| 35 | 3.571 | 3.893 | 4.196 |
| 36 | 3.509 | 3.843 | 4.133 |
| 37 | 3.465 | 3.838 | 4.106 |
| 38 | 3.401 | 3.816 | 4.104 |
| 39 | 3.383 | 3.740 | 4.075 |
| 40 | 3.349 | 3.723 | 4.034 |
| 41 | 3.345 | 3.712 | 4.008 |
| 42 | 3.313 | 3.710 | 4.000 |
| 43 | 3.265 | 3.708 | 3.994 |
| 44 | 3.062 | 3.693 | 3.944 |
| 45 | 2.982 | 3.675 | 3.887 |
| 46 | 2.965 | 3.637 | 3.887 |
| 47 | 2.964 | 3.611 | 3.816 |
| 48 | 2.943 | 3.611 | 3.780 |
| 49 | 2.943 | 3.564 | 3.735 |
| 50 | 2.932 | 3.558 | 3.695 |
| 51 | 2.806 | 3.551 | 3.648 |
| 52 | 2.781 | 3.525 | 3.578 |
| 53 | 2.780 | 3.494 | 3.542 |
| 54 | 2.741 | 3.492 | 3.518 |
| 55 | 2.679 | 3.470 | 3.510 |
| 56 | 2.604 | 3.455 | 3.492 |
| 57 | 2.570 | 3.449 | 3.457 |
| 58 | 2.568 | 3.421 | 3.456 |
| 59 | 2.568 | 3.280 | 3.349 |
| 60 |  | 3.215 | 3.230 |
| 61 |  | 3.177 | 3.200 |
| 62 |  | 3.148 | 3.151 |
| 63 |  | 3.128 | 3.124 |
| 64 |  | 3.058 | 3.058 |
| 65 |  | 2.935 | 3.053 |
| 66 |  | 2.845 | 3.039 |
| 67 |  | 2.837 | 2.968 |
| 68 |  | 2.807 | 2.779 |
| 69 |  | 2.794 | 2.744 |
| 70 |  | 2.785 | 2.699 |
| 71 |  | 2.769 | 2.315 |
| 72 |  | 2.767 | 2.245 |
| 73 |  | 2.674 | 2.193 |
| 74 |  | 2.562 | 1.716 |
| 75 |  | 2.554 |  |
| 76 |  | 2.521 |  |
| 77 |  | 2.488 |  |
| 78 |  | 2.445 |  |
| 79 |  | 2.183 |  |
| 80 |  | 1.965 |  |

|  | Percentage = (no. of cell in that size / total cells)*100 | | |  |  | error bar=std dev/ SQRT(n=number of reads) |
| --- | --- | --- | --- | --- | --- | --- |
| **Cell size** | **SS1** | **SS2** | **SS3** | **average** | **std dev** | **std error** |
| 1 | 0.00 | 1.25 | 1.35 | 0.867 | 0.753 | 0.43 |
| 2 | 25.42 | 18.75 | 9.46 | 17.878 | 8.018 | 4.63 |
| 3 | 33.90 | 37.50 | 32.43 | 34.610 | 2.608 | 1.51 |
| 4 | 20.34 | 21.25 | 33.78 | 25.124 | 7.513 | 4.34 |
| 5 | 15.25 | 13.75 | 18.92 | 15.974 | 2.659 | 1.53 |
| 6 | 3.39 | 5.00 | 4.05 | 4.148 | 0.809 | 0.47 |
| 7 | 1.69 | 1.25 | 0.00 | 0.982 | 0.879 | 0.51 |
| 8 | 0.00 | 1.25 | 0.00 | 0.417 | 0.722 | 0.42 |

3) Normal *C. zofingiensis*(NZ)

| No. of cells | Diameter of cells (µm) | | |
| --- | --- | --- | --- |
|  | Replicate 1 | Replicate 2 | Replicate 3 |
| 2 | 7.768 | 8.631 | 8.675 |
| 3 | 7.664 | 7.746 | 8.351 |
| 4 | 7.566 | 7.632 | 7.254 |
| 5 | 7.535 | 6.904 | 7.027 |
| 6 | 7.144 | 6.775 | 6.963 |
| 7 | 6.780 | 6.447 | 6.869 |
| 8 | 6.587 | 6.295 | 6.797 |
| 9 | 6.375 | 6.234 | 6.435 |
| 10 | 6.373 | 6.010 | 6.125 |
| 11 | 6.301 | 5.996 | 6.083 |
| 12 | 6.049 | 5.943 | 5.991 |
| 13 | 5.816 | 5.930 | 5.944 |
| 14 | 5.805 | 5.711 | 5.585 |
| 15 | 5.685 | 5.696 | 5.574 |
| 16 | 5.537 | 5.681 | 5.494 |
| 17 | 5.465 | 5.637 | 5.434 |
| 18 | 5.453 | 5.603 | 5.431 |
| 19 | 5.366 | 5.534 | 5.383 |
| 20 | 5.358 | 5.534 | 5.379 |
| 21 | 5.315 | 5.399 | 5.217 |
| 22 | 5.301 | 5.328 | 4.990 |
| 23 | 5.274 | 5.243 | 4.985 |
| 24 | 5.183 | 5.174 | 4.942 |
| 25 | 5.158 | 5.130 | 4.818 |
| 26 | 5.142 | 5.084 | 4.754 |
| 27 | 5.060 | 4.986 | 4.667 |
| 28 | 5.054 | 4.952 | 4.652 |
| 29 | 4.992 | 4.922 | 4.591 |
| 30 | 4.974 | 4.892 | 4.557 |
| 31 | 4.936 | 4.887 | 4.314 |
| 32 | 4.915 | 4.635 | 4.215 |
| 33 | 4.877 | 4.590 | 4.069 |
| 34 | 4.864 | 4.583 | 4.049 |
| 35 | 4.835 | 4.573 | 3.794 |
| 36 | 4.818 | 4.573 | 3.794 |
| 37 | 4.779 | 4.541 | 3.757 |
| 38 | 4.631 | 4.365 | 3.751 |
| 39 | 4.589 | 4.168 | 3.615 |
| 40 | 4.586 | 4.119 | 3.530 |
| 41 | 4.482 | 4.083 | 3.507 |
| 42 | 4.460 | 3.938 | 3.489 |
| 43 | 4.399 | 3.878 | 3.446 |
| 44 | 4.344 | 3.801 | 3.425 |
| 45 | 4.261 | 3.776 | 3.412 |
| 46 | 4.166 | 3.714 | 3.398 |
| 47 | 4.114 | 3.673 | 3.388 |
| 48 | 3.982 | 3.580 | 3.317 |
| 49 | 3.953 | 3.555 | 3.288 |
| 50 | 3.918 | 3.423 | 3.240 |
| 51 | 3.851 | 3.400 | 3.201 |
| 52 | 3.469 | 3.367 | 3.184 |
| 53 | 3.330 | 3.347 | 3.182 |
| 54 | 3.322 | 3.273 | 3.162 |
| 55 | 3.212 | 3.217 | 3.152 |
| 56 | 3.152 | 3.062 | 3.105 |
| 57 | 2.699 | 3.059 | 3.091 |
| 58 | 2.682 | 2.999 | 3.051 |
| 59 |  | 2.945 | 3.008 |
| 60 |  | 2.888 | 2.914 |
| 61 |  | 2.677 | 2.914 |
| 62 |  | 2.666 | 2.892 |
| 63 |  | 2.630 | 2.881 |
| 64 |  | 2.583 | 2.852 |
| 65 |  | 2.363 | 2.790 |
| 66 |  |  | 2.768 |
| 67 |  |  | 2.717 |
| 68 |  |  | 2.631 |
| 69 |  |  | 2.606 |
| 70 |  |  | 2.574 |
| 71 |  |  | 2.462 |
| 72 |  |  | 2.384 |
| 73 |  |  | 2.218 |
| 74 |  |  | 2.188 |
| 75 |  |  | 2.135 |
| 76 |  |  | 1.853 |

|  | Percentage = (no. of cell in that size / total cells)*100 | | |  |  | error bar=std dev/ SQRT(n=number of reads) |
| --- | --- | --- | --- | --- | --- | --- |
| **Cell size** | **NZ1** | **NZ2** | **NZ3** | **average** | **std dev** | **std error** |
| 1 | 0.000 | 0.000 | 1.316 | 0.439 | 0.760 | 0.44 |
| 2 | 3.448 | 12.308 | 21.053 | 12.270 | 8.802 | 5.08 |
| 3 | 15.517 | 24.615 | 32.895 | 24.342 | 8.692 | 5.02 |
| 4 | 32.759 | 23.077 | 17.105 | 24.314 | 7.900 | 4.56 |
| 5 | 27.586 | 24.615 | 13.158 | 21.786 | 7.619 | 4.40 |
| 6 | 10.345 | 9.231 | 7.895 | 9.157 | 1.227 | 0.71 |
| 7 | 8.621 | 3.077 | 2.632 | 4.776 | 3.337 | 1.93 |
| 8 | 1.724 | 1.538 | 2.632 | 1.965 | 0.585 | 0.34 |
| 9 | 0.000 | 0.000 | 0.000 | 0.000 | 0.000 | 0.00 |
| 10 | 0.000 | 1.538 | 0.000 | 0.513 | 0.888 | 0.51 |
| 11 | 0.000 | 0.000 | 1.316 | 0.439 | 0.760 | 0.44 |

4) Stress *C. zofingiensis*(SZ)

| No. of cells | Diameter of cells (µm) | | |
| --- | --- | --- | --- |
|  | Replicate 1 | Replicate 2 | Replicate 3 |
| 1 | 13.217 | 14.283 | 15.168 |
| 2 | 12.686 | 11.553 | 13.881 |
| 3 | 12.552 | 11.544 | 13.280 |
| 4 | 12.181 | 11.509 | 13.117 |
| 5 | 11.320 | 11.084 | 11.608 |
| 6 | 10.635 | 10.967 | 11.588 |
| 7 | 10.564 | 10.887 | 11.006 |
| 8 | 10.492 | 10.540 | 10.650 |
| 9 | 9.245 | 10.387 | 10.596 |
| 10 | 9.064 | 10.349 | 10.271 |
| 11 | 9.052 | 10.175 | 10.020 |
| 12 | 8.983 | 9.570 | 9.866 |
| 13 | 8.919 | 9.262 | 9.678 |
| 14 | 8.413 | 8.982 | 9.611 |
| 15 | 8.266 | 8.909 | 9.532 |
| 16 | 8.043 | 8.239 | 9.406 |
| 17 | 8.007 | 8.159 | 9.255 |
| 18 | 7.892 | 8.059 | 9.228 |
| 19 | 7.722 | 7.795 | 9.165 |
| 20 | 7.433 | 7.703 | 8.928 |
| 21 | 7.393 | 7.668 | 8.698 |
| 22 | 7.313 | 7.654 | 8.568 |
| 23 | 7.160 | 7.590 | 8.560 |
| 24 | 7.128 | 7.565 | 8.387 |
| 25 | 6.667 | 7.436 | 7.875 |
| 26 | 6.480 | 7.374 | 7.737 |
| 27 | 6.476 | 7.288 | 7.554 |
| 28 | 6.463 | 6.828 | 7.478 |
| 29 | 6.430 | 6.679 | 7.281 |
| 30 | 6.409 | 6.658 | 6.927 |
| 31 | 6.363 | 6.615 | 6.876 |
| 32 | 6.363 | 6.266 | 6.851 |
| 33 | 6.339 | 6.154 | 6.737 |
| 34 | 6.260 | 6.083 | 6.677 |
| 35 | 6.193 | 6.077 | 6.471 |
| 36 | 6.193 | 6.023 | 6.307 |
| 37 | 6.133 | 6.011 | 6.272 |
| 38 | 6.015 | 5.790 | 6.172 |
| 39 | 5.784 | 5.785 | 6.077 |
| 40 | 5.694 | 5.696 | 5.787 |
| 41 | 5.575 | 5.562 | 5.685 |
| 42 | 5.533 | 5.358 | 5.555 |
| 43 | 5.512 | 5.322 | 5.537 |
| 44 | 5.460 | 5.164 | 5.488 |
| 45 | 5.088 | 5.148 | 5.437 |
| 46 | 5.067 | 4.987 | 5.405 |
| 47 | 5.014 | 4.943 | 5.320 |
| 48 | 4.915 | 4.912 | 5.313 |
| 49 | 4.532 | 4.900 | 5.308 |
| 50 | 4.469 | 4.859 | 5.072 |
| 51 | 4.428 | 4.769 | 4.990 |
| 52 | 4.073 | 4.584 | 4.897 |
| 53 | 3.952 | 4.578 | 4.689 |
| 54 | 3.774 | 4.531 | 4.568 |
| 55 | 3.357 | 4.479 | 4.327 |
| 56 |  | 4.472 | 4.031 |
| 57 |  | 4.406 | 3.352 |
| 58 |  | 4.375 |  |
| 59 |  | 4.346 |  |
| 60 |  | 4.297 |  |
| 61 |  | 4.254 |  |
| 62 |  | 4.251 |  |
| 63 |  | 4.251 |  |
| 64 |  | 4.209 |  |
| 65 |  | 4.190 |  |
| 66 |  | 4.166 |  |
| 67 |  | 4.065 |  |
| 68 |  | 3.916 |  |
| 69 |  | 3.886 |  |
| 70 |  | 3.876 |  |
| 71 |  | 3.841 |  |
| 72 |  | 3.810 |  |
| 73 |  | 3.789 |  |
| 74 |  | 3.483 |  |
| 75 |  | 3.471 |  |
| 76 |  | 3.385 |  |
| 77 |  | 3.297 |  |
| 78 |  | 3.294 |  |

|  | Percentage = (no. of cell in that size / total cells)*100 | | |  |  | error bar=std dev/ SQRT(n=number of reads) |
| --- | --- | --- | --- | --- | --- | --- |
| **Cell size** | **SZ1** | **SZ2** | **SZ3** | **average** | **std dev** | **std error** |
| 1 | 0 | 0 | 0 | 0.000 | 0.000 | 0.00 |
| 2 | 0 | 0 | 0 | 0.000 | 0.000 | 0.00 |
| 3 | 5.45 | 14.10 | 1.75 | 7.104 | 6.337 | 3.66 |
| 4 | 9.09 | 28.21 | 10.53 | 15.941 | 10.645 | 6.15 |
| 5 | 16.36 | 10.26 | 19.30 | 15.306 | 4.613 | 2.66 |
| 6 | 25.45 | 12.82 | 17.54 | 18.606 | 6.384 | 3.69 |
| 7 | 12.73 | 11.54 | 8.77 | 11.013 | 2.029 | 1.17 |
| 8 | 10.91 | 6.41 | 8.77 | 8.697 | 2.250 | 1.30 |
| 9 | 5.45 | 2.56 | 14.04 | 7.351 | 5.966 | 3.44 |
| 10 | 5.45 | 7.69 | 7.02 | 6.721 | 1.148 | 0.66 |
| 11 | 1.82 | 5.13 | 5.26 | 4.070 | 1.951 | 1.13 |
| 12 | 5.45 | 0.00 | 0.00 | 1.818 | 3.149 | 1.82 |
| 13 | 1.82 | 0.00 | 5.26 | 2.360 | 2.673 | 1.54 |
| 14 | 0.00 | 1.28 | 0.00 | 0.427 | 0.740 | 0.43 |
| 15 | 0.00 | 0.00 | 1.75 | 0.585 | 1.013 | 0.58 |

**Fig. 5** Total phenolic content of *C. sorokiniana* and *C. zofingiensis* under normal and stress conditions.

| **Blank** | **a** | **b** | **c** | **average** |
| --- | --- | --- | --- | --- |
| dH2O+reagent | 0.057 | 0.056 | 0.057 | 0.056667 |

| **Gallic acid**  **(µg/mL)** | **a** | **b** | **c** | **average** | **av-blank** | **std dev** |
| --- | --- | --- | --- | --- | --- | --- |
| 1.563 | 0.061 | 0.062 | 0.062 | 0.061 | 0.005 | 0.000 |
| 3.125 | 0.060 | 0.060 | 0.060 | 0.060 | 0.003 | 0.000 |
| 6.250 | 0.063 | 0.063 | 0.064 | 0.063 | 0.007 | 0.001 |
| 12.500 | 0.069 | 0.069 | 0.070 | 0.069 | 0.013 | 0.001 |
| 25.000 | 0.079 | 0.079 | 0.080 | 0.079 | 0.023 | 0.000 |
| 50.000 | 0.098 | 0.098 | 0.099 | 0.098 | 0.041 | 0.001 |
| 100.000 | 0.130 | 0.191 | 0.131 | 0.151 | 0.094 | 0.035 |
| 200.000 | 0.226 | 0.238 | 0.232 | 0.232 | 0.175 | 0.006 |

| **Control = sample + dH20** | **mg/mL** | **a** | **b** | **c** | **average** |
| --- | --- | --- | --- | --- | --- |
| Normal *C. sorokiniana* | 4 | 0.12 | 0.12 | 0.12 | 0.12 |
| Normal *C. zofingiensis* | 4 | 0.211 | 0.211 | 0.211 | 0.211 |
| Stress *C. sorokiniana* | 4 | 0.098 | 0.098 | 0.099 | 0.098 |
| Stress *C zofingiensis* | 4 | 0.08 | 0.08 | 0.08 | 0.08 |

|  |  |  |  |  | **Absorbance-control** | | | **Gallic acid equivalent** | | | **µg gallic acid/mg sample** | | |  |  |
| --- | --- | --- | --- | --- | --- | --- | --- | --- | --- | --- | --- | --- | --- | --- | --- |
|  |  |  |  |  |  |  |  | **y = 1139x - 1.5945** | | |  |  |  |  |  |
| **Sample** | **mg/mL** | **a** | **b** | **c** | **a** | **b** | **c** | **a** | **b** | **c** | **a** | **b** | **c** | **average** | **std dev** |
| Normal *C. sorokiniana* | 4 | 0.378 | 0.381 | 0.381 | 0.258 | 0.261 | 0.261 | 292.647 | 295.685 | 296.064 | 73.162 | 73.921 | 74.016 | 73.700 | 0.468 |
| Normal *C. zofingiensis* | 4 | 0.355 | 0.356 | 0.357 | 0.144 | 0.145 | 0.146 | 162.042 | 163.561 | 164.700 | 40.510 | 40.890 | 41.175 | 40.858 | 0.333 |
| Stress *C. sorokiniana* | 4 | 0.139 | 0.141 | 0.141 | 0.041 | 0.042 | 0.043 | 45.105 | 46.623 | 47.003 | 11.276 | 11.656 | 11.751 | 11.561 | 0.251 |
| Stress *C. zofingiensis* | 4 | 0.133 | 0.135 | 0.136 | 0.053 | 0.055 | 0.056 | 58.773 | 60.671 | 61.810 | 14.693 | 15.168 | 15.452 | 15.104 | 0.384 |

**Fig. 6** Percentage of DPPH radical scavenging activity by *C. sorokiniana* and *C. zofingiensis* under normal and stress conditions.

|  |  | **sample** | | | **control =**  **sample + dH2O** | | | **control - sample** | | | **([control-sample]/control)*100** | | | **1 mg/mL** | | |  |  |
| --- | --- | --- | --- | --- | --- | --- | --- | --- | --- | --- | --- | --- | --- | --- | --- | --- | --- | --- |
|  | **mg/mL** | **a** | **b** | **c** | **a** | **b** | **c** | **a** | **b** | **c** | **a** | **b** | **c** | **a** | **b** | **c** | **average** | **std dev** |
| Normal *C. sorokiniana* | 4 | 0.95 | 0.939 | 0.932 | 1.748 | 1.74 | 1.748 | 0.798 | 0.801 | 0.816 | 45.652 | 46.034 | 46.682 | 11.413 | 11.509 | 11.670 | 11.531 | 0.130 |
| Normal *C. zofingiensis* | 4 | 0.735 | 0.727 | 0.724 | 1.502 | 1.511 | 1.531 | 0.767 | 0.784 | 0.807 | 51.065 | 51.886 | 52.711 | 12.766 | 12.972 | 13.178 | 12.972 | 0.206 |
| Stress *C. sorokiniana* | 4 | 0.666 | 0.6615 | 0.6615 | 1.46 | 1.463 | 1.47 | 0.794 | 0.8015 | 0.809 | 54.384 | 54.785 | 55.000 | 13.596 | 13.696 | 13.750 | 13.681 | 0.078 |
| Stress *C. zofingiensis* | 4 | 0.723 | 0.721 | 0.719 | 1.609 | 1.635 | 1.668 | 0.886 | 0.914 | 0.949 | 55.065 | 55.902 | 56.894 | 13.766 | 13.976 | 14.224 | 13.988 | 0.229 |

**Fig. 7** Ferric reducing antioxidant power of *C. sorokiniana* and *C. zofingiensis* under normal and stress condition.

| **Ascorbic acid** |  |  |  |  |  |
| --- | --- | --- | --- | --- | --- |
| **µg/mL** | **a** | **b** | **c** | **average** | **std dev** |
| 1.563 | 0.247 | 0.247 | 0.248 | 0.247 | 0.001 |
| 3.125 | 0.280 | 0.281 | 0.281 | 0.281 | 0.001 |
| 6.25 | 0.470 | 0.472 | 0.473 | 0.472 | 0.002 |
| 12.5 | 0.857 | 0.857 | 0.860 | 0.858 | 0.002 |
| 25 | 1.613 | 1.620 | 1.620 | 1.618 | 0.004 |
| 50 | 2.850 | 2.851 | 2.860 | 2.854 | 0.006 |
| 100 | 3.696 | 3.668 | 3.605 | 3.656 | 0.047 |
| 200 | 3.642 | 3.590 | 3.534 | 3.588 | 0.054 |

|  |  |  |  |  |  |  |  |  |  |  | ascorbic acid equivalent | | | µg ascorbic acid/mg sample | | |  |  |
| --- | --- | --- | --- | --- | --- | --- | --- | --- | --- | --- | --- | --- | --- | --- | --- | --- | --- | --- |
|  |  | sample | | | Control= sample + dH20 | | | Absorbance-control | | | y = 18.181x - 2.7714 | | | (ascorbic acid µg/mL / sample mg/mL) | | |  |  |
|  | mg/mL | a | b | c | a | b | c | a | b | c | a | b | c | a | b | c | average | std dev |
| Normal *C. sorokiniana* | 4 | 1.746 | 1.714 | 1.706 | 0.217 | 0.221 | 0.222 | 1.529 | 1.493 | 1.484 | 25.033 | 24.367 | 24.209 | 6.258 | 6.092 | 6.052 | 6.134 | 0.109 |
| Normal *C. zofingiensis* | 4 | 2.398 | 2.396 | 2.386 | 0.195 | 0.196 | 0.195 | 2.203 | 2.200 | 2.191 | 37.275 | 37.221 | 37.057 | 9.319 | 9.305 | 9.264 | 9.296 | 0.028 |
| Stress *C. sorokiniana* | 4 | 0.501 | 0.498 | 0.498 | 0.117 | 0.114 | 0.113 | 0.384 | 0.384 | 0.385 | 4.204 | 4.210 | 4.228 | 1.051 | 1.053 | 1.057 | 1.054 | 0.003 |
| Stress *C. zofingiensis* | 4 | 0.457 | 0.462 | 0.466 | 0.122 | 0.119 | 0.118 | 0.335 | 0.343 | 0.348 | 3.313 | 3.459 | 3.550 | 0.828 | 0.865 | 0.887 | 0.860 | 0.030 |

**Table 1** Pigments present in the microalgae *C. sorokiniana* and *C. zofingiensis* under normal and stress conditions.

Note:

NS: Normal *C. sorokiniana*

NZ: Normal *C. zofingiensis*

SS: Stress *C. sorokiniana*

SZ: Stress *C. zofingiensis*

df: dilution factor

Abs: Absorbance

1) Chlorophyll a:

| Replicate 1 | |  |  |  |  |  |  | **chlorophyl a (µg/mL)** |
| --- | --- | --- | --- | --- | --- | --- | --- | --- |
| **Sample** | **mg/mL** | **df** | **Abs at 664** | **Abs at 648** | **Abs at 470** | **Chlorophyll a** | **x df** | **in 1 mg/mL sample** |
| NS | 1 | 2 | 0.807 | 0.355 | 0.739 | 8.939 | 17.878 | 17.878 |
| NZ | 1 | 2 | 0.721 | 0.345 | 0.844 | 7.842 | 15.684 | 15.684 |
| SS | 1 | 2 | 0.118 | 0.055 | 0.655 | 1.291 | 2.582 | 2.582 |
| SZ | 1 | 2 | 0.119 | 0.06 | 0.715 | 1.278 | 2.557 | 2.557 |
|  |  |  |  |  |  |  |  |  |
|  |  |  |  |  |  |  |  |  |
| Replicate 2 | |  |  |  |  |  |  | **chlorophyl a (µg/mL)** |
| **Sample** | **mg/mL** | **df** | **Abs at 664** | **Abs at 648** | **Abs at 470** | **Chlorophyll a** | **x df** | **in 1 mg/mL sample** |
| NS | 1 | 2 | 0.81 | 0.357 | 0.738 | 8.969 | 17.938 | 17.938 |
| NZ | 1 | 2 | 0.722 | 0.347 | 0.832 | 7.845 | 15.690 | 15.690 |
| SS | 1 | 2 | 0.119 | 0.056 | 0.615 | 1.299 | 2.598 | 2.598 |
| SZ | 1 | 2 | 0.123 | 0.062 | 0.715 | 1.322 | 2.643 | 2.643 |
|  |  |  |  |  |  |  |  |  |
|  |  |  |  |  |  |  |  |  |
|  |  |  |  |  |  |  |  |  |
| Replicate 3 | |  |  |  |  |  |  | **chlorophyl a (µg/mL)** |
| **Sample** | **mg/mL** | **df** | **Abs at 664** | **Abs at 648** | **Abs at 470** | **Chlorophyll a** | **x df** | **in 1 mg/mL sample** |
| NS | 1 | 2 | 0.812 | 0.359 | 0.74 | 8.985 | 17.970 | 17.970 |
| NZ | 1 | 2 | 0.723 | 0.349 | 0.841 | 7.848 | 15.696 | 15.696 |
| SS | 1 | 2 | 0.119 | 0.056 | 0.656 | 1.299 | 2.598 | 2.598 |
| SZ | 1 | 2 | 0.122 | 0.064 | 0.726 | 1.298 | 2.596 | 2.596 |

2) Chlorophyll b:

| Replicate 1 | |  |  |  |  |  |  | **chlorophyl b (µg/mL)** |
| --- | --- | --- | --- | --- | --- | --- | --- | --- |
| **Sample** | **mg/mL** | **df** | **Abs at 664** | **Abs at 648** | **Abs at 470** | **Chlorophyll b** | **x df** | **in 1 mg/mL sample** |
| NS | 1 | 2 | 0.807 | 0.355 | 0.739 | 3.185 | 6.370 | 6.370 |
| NZ | 1 | 2 | 0.721 | 0.345 | 0.844 | 3.609 | 7.218 | 7.218 |
| SS | 1 | 2 | 0.118 | 0.055 | 0.655 | 0.550 | 1.101 | 1.101 |
| SZ | 1 | 2 | 0.119 | 0.06 | 0.715 | 0.680 | 1.359 | 1.359 |
|  |  |  |  |  |  |  |  |  |
| Replicate 2 | |  |  |  |  |  |  | **chlorophyl b (µg/mL)** |
| **Sample** | **mg/mL** | **df** | **Abs at 664** | **Abs at 648** | **Abs at 470** | **Chlorophyll b** | **x df** | **in 1 mg/mL sample** |
| NS | 1 | 2 | 0.81 | 0.357 | 0.738 | 3.215 | 6.431 | 6.431 |
| NZ | 1 | 2 | 0.722 | 0.347 | 0.832 | 3.656 | 7.311 | 7.311 |
| SS | 1 | 2 | 0.119 | 0.056 | 0.615 | 0.570 | 1.140 | 1.140 |
| SZ | 1 | 2 | 0.123 | 0.062 | 0.715 | 0.702 | 1.404 | 1.404 |
|  |  |  |  |  |  |  |  |  |
| Replicate 3 | |  |  |  |  |  |  | **chlorophyl b (µg/mL)** |
| **Sample** | **mg/mL** | **df** | **Abs at 664** | **Abs at 648** | **Abs at 470** | **Chlorophyll b** | **x df** | **in 1 mg/mL sample** |
| NS | 1 | 2 | 0.812 | 0.359 | 0.74 | 3.254 | 6.508 | 6.508 |
| NZ | 1 | 2 | 0.723 | 0.349 | 0.841 | 3.702 | 7.405 | 7.405 |
| SS | 1 | 2 | 0.119 | 0.056 | 0.656 | 0.570 | 1.140 | 1.140 |
| SZ | 1 | 2 | 0.122 | 0.064 | 0.726 | 0.765 | 1.530 | 1.530 |

3) Total carotene:

| Replicate 1 | |  |  |  |  |  |  |  |  |  | **total carotene (µg/mL)** |
| --- | --- | --- | --- | --- | --- | --- | --- | --- | --- | --- | --- |
| **Sample** | **mg/mL** | **df** | **Abs at 664** | **Abs at 648** | **Abs at 470** | **Chlorophyll a** | **Chlorophyll b** | **carotene** | **total carotene** | **x df** | **in 1 mg/mL sample** |
| NS | 1 | 2 | 0.807 | 0.355 | 0.739 | 8.939 | 3.185 | 408.995 | 1.957 | 3.914 | 3.914 |
| NZ | 1 | 2 | 0.721 | 0.345 | 0.844 | 7.842 | 3.609 | 452.435 | 2.047 | 4.094 | 4.094 |
| SS | 1 | 2 | 0.118 | 0.055 | 0.655 | 1.291 | 0.550 | 595.116 | 2.693 | 5.386 | 5.386 |
| SZ | 1 | 2 | 0.119 | 0.06 | 0.715 | 1.278 | 0.680 | 641.594 | 2.903 | 5.806 | 5.806 |
|  |  |  |  |  |  |  |  |  |  |  |  |
| Replicate 2 | |  |  |  |  |  |  |  |  |  | **total carotene (µg/mL)** |
| **Sample** | **mg/mL** | **df** | **Abs at 664** | **Abs at 648** | **Abs at 470** | **Chlorophyll a** | **Chlorophyll b** | **carotene** | **total carotene** | **x df** | **in 1 mg/mL sample** |
| NS | 1 | 2 | 0.81 | 0.357 | 0.738 | 8.969 | 3.215 | 404.954 | 1.938 | 3.875 | 3.875 |
| NZ | 1 | 2 | 0.722 | 0.347 | 0.832 | 7.845 | 3.656 | 435.524 | 1.971 | 3.941 | 3.941 |
| SS | 1 | 2 | 0.119 | 0.056 | 0.615 | 1.299 | 0.570 | 553.076 | 2.503 | 5.005 | 5.005 |
| SZ | 1 | 2 | 0.123 | 0.062 | 0.715 | 1.322 | 0.702 | 639.175 | 2.892 | 5.784 | 5.784 |
|  |  |  |  |  |  |  |  |  |  |  |  |
| Replicate 3 | |  |  |  |  |  |  |  |  |  | **total carotene (µg/mL)** |
| **Sample** | **mg/mL** | **df** | **Abs at 664** | **Abs at 648** | **Abs at 470** | **Chlorophyll a** | **Chlorophyll b** | **carotene** | **total carotene** | **x df** | **in 1 mg/mL sample** |
| NS | 1 | 2 | 0.812 | 0.359 | 0.74 | 8.985 | 3.254 | 403.148 | 1.929 | 3.858 | 3.858 |
| NZ | 1 | 2 | 0.723 | 0.349 | 0.841 | 7.848 | 3.702 | 439.613 | 1.989 | 3.978 | 3.978 |
| SS | 1 | 2 | 0.119 | 0.056 | 0.656 | 1.299 | 0.570 | 594.076 | 2.688 | 5.376 | 5.376 |
| SZ | 1 | 2 | 0.122 | 0.064 | 0.726 | 1.298 | 0.765 | 643.603 | 2.912 | 5.824 | 5.824 |
